# A digital pathway for genetic testing in UK NHS patients with cancer: BRCA-DIRECT randomised study internal pilot

**DOI:** 10.1136/jmg-2022-108655

**Published:** 2022-07-22

**Authors:** Bethany Torr, Christopher Jones, Subin Choi, Sophie Allen, Grace Kavanaugh, Monica Hamill, Alice Garrett, Suzanne MacMahon, Lucy Loong, Alistair Reay, Lina Yuan, Mikel Valganon Petrizan, Kathryn Monson, Nicky Perry, Lesley Fallowfield, Valerie Jenkins, Rochelle Gold, Amy Taylor, Rhian Gabe, Jennifer Wiggins, Anneke Lucassen, Ranjit Manchanda, Ashu Gandhi, Angela George, Michael Hubank, Zoe Kemp, D Gareth Evans, Stephen Bremner, Clare Turnbull

**Affiliations:** 1 Institute of Cancer Research, Division of Genetics and Epidemiology, Sutton, UK; 2 Clinical Trials Unit, Brighton and Sussex Medical School, Brighton, UK; 3 Centre for Molecular Pathology, Institute of Cancer Research Sutton, Sutton, UK; 4 Sussex Health Outcomes, Research and Education in Cancer (SHORE-C), Brighton and Sussex Medical School, Brighton, UK; 5 BRCA Journey, Patient Representative, Leeds, UK; 6 Clinical Genetics, East Anglian Medical Genetics Service, Cambridge, UK; 7 Wolfson Institute of Population Health, Queen Mary’s University of London, London, UK; 8 Cancer Genetics Unit, Royal Marsden NHS Foundation Trust, London, UK; 9 Clinical Ethics and Law at Southampton (CELS), University of Southampton, Southampton, UK; 10 Department of Medicine, Univerity of Oxford Nuffield, Oxford, UK; 11 Department of Gynaecological Oncology, Barts Health NHS Trust, London, UK; 12 Department of Health Services Research, Faculty of Public Health & Policy, London School of Hygiene and Tropical Medicine, London, UK; 13 School of Cancer Sciences, Faculty of Biology, Medicine and Health, University of Manchester, Manchester Academic Health Science Centre, Manchester, UK; 14 Prevent Breast Cancer Centre, Wythenshawe Hospital Manchester Universities Foundation Trust, Manchester, UK; 15 Nightingale and Genesis Breast Cancer Centre, University Hospital of South Manchester NHS Foundation Trust, Manchester, UK; 16 Division of Evolution and Genomic Sciences, The University of Manchester, Manchester, UK

**Keywords:** genetics, genetic counseling, genetic predisposition to disease, genetic testing, health care facilities, manpower, and services

## Abstract

**Background:**

Germline genetic testing affords multiple opportunities for women with breast cancer, however, current UK NHS models for delivery of germline genetic testing are clinician-intensive and only a minority of breast cancer cases access testing.

**Methods:**

We designed a rapid, digital pathway, supported by a genetics specialist hotline, for delivery of germline testing of *BRCA1/BRCA2/PALB2* (BRCA-testing), integrated into routine UK NHS breast cancer care. We piloted the pathway, as part of the larger BRCA-DIRECT study, in 130 unselected patients with breast cancer and gathered preliminary data from a randomised comparison of delivery of pretest information digitally (fully digital pathway) or via telephone consultation with a genetics professional (partially digital pathway).

**Results:**

Uptake of genetic testing was 98.4%, with good satisfaction reported for both the fully and partially digital pathways. Similar outcomes were observed in both arms regarding patient knowledge score and anxiety, with <5% of patients contacting the genetics specialist hotline. All progression criteria established for continuation of the study were met.

**Conclusion:**

Pilot data indicate preliminary demonstration of feasibility and acceptability of a fully digital pathway for BRCA-testing and support proceeding to a full powered study for evaluation of non-inferiority of the fully digital pathway, detailed quantitative assessment of outcomes and operational economic analyses.

**Trial registration number:**

ISRCTN87845055.

**What is already known on this topic**

- Digital alternatives to current appointment-based genetic testing pathways and counselling have been shown to be acceptable in certain patient populations in the UK and internationally, however, acceptability, and effectivity within UK NHS oncology clinics is not well explored.

**What this study adds**

- This study offers preliminary evidence of patient and healthcare professional satisfaction with a digital pathway for NHS diagnostic genetic testing in unselected patients with breast cancer in NHS oncology care and indicates safety and effectiveness of trialling digital information giving versus appointment-based counselling.

**How this study might affect research, practice or policy**

- Findings support additional evaluation of the BRCA-DIRECT digital pathway with greater power.
- The current data and a larger evaluation will potentially be pivotal in generating evidence for the use of digital technologies as a mechanism for expanding genetic testing to more patients with cancer diagnoses within NHS oncology clinics.
- These activities are highly concordant with NHS England’s long-term plan for increased identification of individuals at elevated genetic risk of cancer and increased focus for embedding digital technologies within the NHS.

## Introduction

Testing of patients with cancer for high penetrance breast-ovarian cancer susceptibility gene (CSGs) *BRCA1, BRCA2* and *PALB2* offers three potential benefits. First, identification of a germline pathogenic variant can provide insights into the oncogenesis of their cancer, potentially informing selection of chemotherapeutic agents, including platinum and targeted therapies such as poly-ADP ribose polymerase inhibitors. Second, knowledge of elevated risk of subsequent breast, ovarian and other cancers may direct the patient towards risk-reducing surgery and/or intensive surveillance. Third, cascade testing for the pathogenic variant enables identification of family members who carry it, are at elevated risk of cancer and might benefit from medical interventions, as well as providing reassurance for those who do not carry the familial variant.^1^

Historically, variant scanning along the length of a CSG was labour-intensive and thus expensive, typically taking several months in a diagnostic laboratory. Hence, germline genetic testing was largely divorced from acute diagnostic oncology, instead being initiated more typically by those who had successfully completed treatment for a prior cancer diagnosis and unaffected relatives concerned by their family history. With the advent of next-generation sequencing (NGS) technologies, sequencing of CSGs has become relatively cheap, rapid and high-throughput. Furthermore, we have good knowledge of the pathogenicity of variants in well-characterised genes such as *BRCA1, BRCA2, MLH1* and *MSH2*, such that interpretation can be streamlined via automated bioinformatics pipelines, with a low rate of variants of uncertain significance (VUS). Thus, it is increasingly feasible from the laboratory perspective that large-scale, rapid CSG analysis could be offered routinely as part of the diagnostic workup for all patients with relevant cancers. This in principle offers opportunity for patient’s primary surgery to combine treatment of the current cancer with risk reduction for future cancers. For example, bilateral mastectomy rather than localised excision may be performed in a *BRCA1*-positive woman with newly diagnosed breast cancer.

However, while technological advances have driven massive improvement in laboratory capacity, substantial barriers within the upstream and downstream clinical pathways remain. The traditional model of individualised patient referral to clinical genetics for management of pretest genetic counselling, consenting, sample acquisition and return of results reflects an era in which patient volumes were low and timescales unpressured. To facilitate rapid delivery to larger populations of patients with cancer, there have been attempts to transition germline genetic testing across into mainstream oncology.^2–4^ For ovarian cancer, of which there are ~7500 cases per year in the UK and a ~15% frequency of germline pathogenic variants of *BRCA1/BRCA2*,^5 6^ over the last 5 years there has been relatively successful implementation of universal testing using a variety of ‘mainstreaming’ models.^2 7^ For breast cancer, the incidence is much higher (~56 000/year)^8^ while the pick-up rate of pathogenic variants is more modest (~3%–5% in total for *BRCA1/BRCA2/PALB2*).^9–11^ Delivery of germline genetic testing by mainstream breast cancer oncological clinicians has been piloted, but success has been more limited. Lack of requisite expertise, high workload, large patient numbers and perceived relevance of germline testing for immediate clinical decision-making have been cited as causes for clinician reluctance.^12^

Limitations of the precision oncology model in improving outcomes in those diagnosed with advanced cancers has led to renewed focus on improving cancer early detection and prevention. Such interventions are most impactful applied to those at very high priori risk of cancer. However; despite being the earliest, most common and arguably most widely established paradigm of high penetrance cancer susceptibility, recent analyses suggest low ascertainment, with fewer than 3% of BRCA1/BRCA2 heterozygotes across Greater London identified.^13^

Recent health economic analyses, using UK-specific and other costing parameters, demonstrate testing of *BRCA1/BRCA2* genes in unselected patients with breast cancer to be cost-effective.^14–17^ However, the current NHS eligibility criteria exclude >80% of patients with breast cancer from germline genetic testing, a restriction now arguably driven more by capacity and costs of clinical manpower than laboratory assays.^18–20^ It is paradoxical therefore, that complex evaluation of family history to exclude ineligible patients still occupies a substantial proportion of capacity of expert genetics clinicians of which the system is so short. Furthermore, due to small family size, male transmission, fractured transmission of familial information and variation in penetrance and chance, the current family based criteria fail to catch about half of BRCA heterozygotes.^21 22^

An additional consequence of this high threshold for NHS germline genetic testing is diversion of ‘ineligible’ patients with breast cancer to private and direct-to-consumer testing. As well as driving inequity, these laboratories function outside of the regulatory standards and informatics systems unifying the UK NHS diagnostic laboratory network, through which we ensure that variant information and classifications are consistent, shared and updated. These parallel systems have ethical implications regarding equity of access to downstream NHS-funded interventions,^23 24^ and create additional friction regarding NHS evaluation of spurious results for patients who were ineligible for NHS testing.^24 25^

We and others have hypothesised that integration within NHS diagnostic cancer pathways of simple technology platforms could mitigate the impasse.^26–28^ Requirement for detailed individualised genetic counselling to inform the decision to undertake a genetic test comes from early models for Huntington disease and prenatal scenarios.^29^ For a cancer patient, a germline genetic test is arguably one component of a suite of tests potentially informing their cancer management, and while there are also important considerations to be made about possible implications of the result for their future and family, the information relating to the test is largely generic. Likewise, operational management of consent, sample transmission and return of results is largely formulaic. However, integration within NHS clinical and laboratory information systems of this digital pathway is critical to ensure (i) full communication of results across clinical organisations, (ii) appropriate expert management of nuanced scenarios such as uncertain variants and BRCA-negative patients with strong/unusual family histories and (iii) ongoing VUS review with patient recontact.

Over the last decade, there has been considerable focus in NHS strategy on application of digital solutions to extend and improve access to healthcare, a trend dramatically catalysed by the COVID-19 pandemic. Furthermore, there has been concurrent high priority within NHS England for expanded application of genetic testing for personalised prediction.^30 31^ We therefore sought to design a digital pathway that was rapid, patient-centred, ‘light-touch’ for clinicians, and integrated into NHS clinical, laboratory and informatics systems, by which genetic testing could be delivered to mainstream patients with cancer at potentially much greater scale. For our exemplar clinical scenario, we applied this pathway to BRCA-testing (*BRCA1/BRCA2/PALB2* gene testing) in unselected mainstream patients with breast cancer.

While a digital pathway would be presumed to improve capacity and efficiency, questions remain regarding whether a digital route might adversely impact anxiety, satisfaction and/or understanding of the genetic testing process for patients with cancer. Furthermore, while we presuppose that patients with cancer would value the rapidity, convenience and flexibility of a digital pathway, these trade-offs have not been well explored, in particular for UK NHS patients.^28 32 33^ As well as evaluating patient responses to the digital pathway, we also leveraged this opportunity to compare by randomisation two approaches to delivery of pretest information. Half the patients underwent the ‘fully digital pathway’ with pretest information delivered digitally, while half the patients had a pretest telephone consultation with a genetics professional (the ‘partially digital pathway’).

We here present data on development of the BRCA-DIRECT pathway, evaluation of our internal pilot of the BRCA-DIRECT pathway in 130 unselected NHS patients with breast cancer, preliminary data from randomised comparison in this population between digital and telephone consultation delivery of pretest information and assessment of progression criterion established to support continuation of the study to the full recruitment target for well powered analyses of outcomes.

## Methods

**Figure 1:**
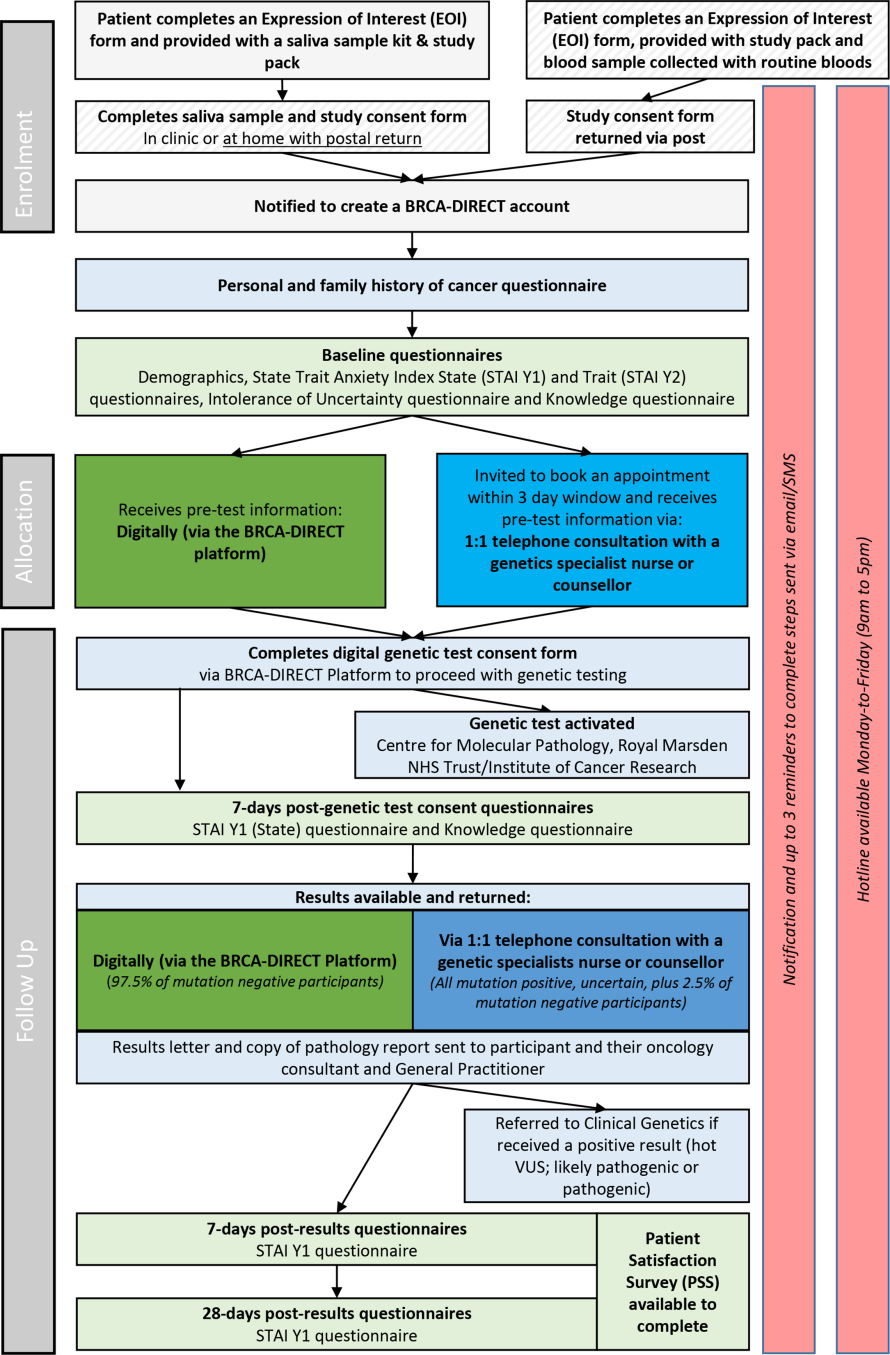
The BRCA-DIRECT full study pathway, including COVID-19 adaptations to minimise aerosol generating procedures (stripped grey). Light grey: study-specific procedures, including enrolment activities and study questionnaires. Light green: BRCA-DIRECT digital pathway core activities. Dark green: fully digital pathway with delivery of pretest information digitally. Blue: partially digital comparator arm with delivery of pretest information via telephone consultation with a genetics professional. VUS, variants of uncertain significance.

We describe materials and methods relating to three constituent activities, namely: (i) development of the BRCA-DIRECT digital pathway, (ii) evaluation of the BRCA-DIRECT digital pathway, (iii) randomised evaluation of delivery pretest information, comparing digital delivery (fully digital pathway) with telephone consultation with a genetics professional (partially digital pathway) as well as (iv) assessment against established progression criteria for continuation of the study (figure 1). For full details, see online supplemental methods.

10.1136/jmg-2022-108655.supp1 Supplementary data

### Design of the BRCA-DIRECT digital pathway

A preliminary pathway and materials were designed by the core study team (three clinical geneticists, two genetic counsellors, one oncology/genetics specialist nurse, five psycho-oncological researchers and two research/study coordinators). This was followed by iterative consultations with (a) a broader clinical group (two clinical geneticists, one genetic counsellor, two oncologists and one oncology surgeon) and (b) a group of eight patients, who evaluated (i) materials for the fully digital pathway for testing of germline *BRCA1/BRCA2/PALB2* and (ii) core functionality of the BRCA-DIRECT digital platform, as described in box 1.

Box 1 **BRCA-DIRECT pathway: key elements**

See also figure 1, online supplemental figure 1.

(1) BRCA-DIRECT telephone hotline:

A telephone hotline staffed by a genetics professional (genetic counsellor, oncogenetics specialist nurse or clinical geneticist) was available 09:00 to 17:00 hours weekdays.

(2) DNA sampling via saliva:

Saliva sample kits and instructions were provided for patients to complete in clinic or at home with postal return.

(3) BRCA-DIRECT digital platform for workflow management and communication to patients:

The BRCA-DIRECT digital platform was accessible via any internet-connected device, with personalised patient and clinical/administrative logins for approved users. Patient workflow was delivered as structured stages, with timed SMS and/or email notifications to alert the patient to next required activity (see online supplemental figure 2).

(4) BRCA-DIRECT digital pretest information:

Digital pretest information, available via the BRCA-DIRECT digital platform, comprised 21 static screens of written information and schematics designed to be equivalent in detail and depth to a standard genetic counselling appointment (online supplemental appendix 1).

(5) Digital genetic test consent:

The genetic test consent form was available to complete digitally via the BRCA-DIRECT platform, following confirmation that the patient had received the pretest information. The digital consent reflected the contents of the ‘Record of Discussion Regarding Genomic Testing’ used in the UK NHS Genomic Medicine Service.

(6) Analysis of *BRCA1/BRCA2/PALB2* (BRCA-testing) in an accredited NHS diagnostic laboratory:

Full analysis of the coding region and intron/exon boundaries of *BRCA1, BRCA2* and *PALB2*, including dosage analysis, was undertaken at The Centre for Molecular Pathology (Royal Marsden NHS Foundation Trust and Institute of Cancer Research, London, UK) accredited to International Organization for Standardization 15189:2012. As per recommendations of UK-ACGS (UK Association of Clinical Genomic Scientists), only variants classified as pathogenic, likely pathogenic or ‘hot’ variants of uncertain significant (hot VUS) (4/5 evidence points) were included on the laboratory report.^34 38 39^

(7) Predominantly digital return of BRCA-test results:

Patients were randomly pre-allocated to receive results digitally (97.5%) or via rapid telephone consultation with a genetics professional (2.5%).^40^ All those with a negative result received their result according to the pre-allocated randomisation. Patients with a reported variant (pathogenic, likely pathogenic, hot VUS (predicted ~5% of patients)) received their result via rapid telephone consultation, regardless of pre-allocated group.

(8) Formal written communication of BRCA-test results to NHS clinicians and follow-up. A summary letter was automatically generated according to the result of negative (no variants reported), positive (pathogenic, likely pathogenic variant reported) or VUS. This letter included the family history as supplied by the patient, along with standardised information on breast surveillance recommendations for BRCA-negative probands and family members. The letter (along with the laboratory report) was sent by post to the patient and general practitioner and by email to the hospital clinical team. For patients in whom a positive result/VUS was reported, an automatically generated referral letter to the local clinical genetics service was also sent.

10.1136/jmg-2022-108655.supp2 Supplementary data

10.1136/jmg-2022-108655.supp3 Supplementary data

### Pilot of the BRCA-DIRECT digital pathway

We piloted the BRCA-DIRECT (fully and partially digital) pathway in 130 unselected patients with breast cancer recruited from three hospital sites under the Royal Marsden NHS Foundation Trust, London, UK (RMH). See figure 1 for full study pathway and adaptations due to COVID-19.

### Eligibility

Patients were eligible if they had a diagnosis of invasive breast cancer or high-grade ductal carcinoma in situ and were above 18 years of age. Inclusion criteria were self-assessed good comprehension of English language, and access to a smartphone and/or email. Exclusions included previous testing for *BRCA1*, *BRCA2* and *PALB2*.

### Study recruitment

Information regarding BRCA-DIRECT was made available in outpatients departments via posters, leaflets and verbally from oncology professionals (specialist nurses and doctors). Those patients expressing interest were provided with a BRCA-DIRECT pack comprising a saliva collection kit, study patient information sheet and consent form and a postal return envelope. On receipt of the study consent form, patients were notified via email and/or SMS and enabled to create an account on the BRCA-DIRECT platform.

### Baseline data collection

Demographic data relevant to *evaluation* of the BRCA-DIRECT pathway (eg, educational status, level of social support, ethnicity) were collected digitally via the BRCA-DIRECT platform, in addition to data core to the BRCA-DIRECT pathway (eg, relevant personal and family history of cancer). Key medical details were confirmed by the study team from the patient medical record, including confirmation of cancer status, status of breast cancer treatment and (planned) surgery date.

### Outcomes for evaluation of the BRCA-DIRECT pathway

We evaluated acceptability of the BRCA-DIRECT digital pathway via the following approaches:

1. Patient progression through the BRCA-DIRECT pathway, evaluating percentage uptake of genetic testing, turnaround times for results (time-to-results) and withdrawals.
2. Patient usage of the BRCA-DIRECT telephone hotline calls, assessing volume, timing and content of calls categorised as either (i) genetics specialist or (ii) administrative.
3. Patient satisfaction with the BRCA-DIRECT digital pathway, evaluating using a 15-item study-specific survey, conducted 7 days postreceipt of test result (T2).
4. Healthcare professional (HCP) satisfaction with the BRCA-DIRECT digital pathway compared with standard clinical care pathways via a 10-item study-specific survey.
5. Structured interviews, involving 26 questions completed with 10 patients to capture more detailed feedback on the BRCA-DIRECT pathway.

### Randomised evaluation comparing BRCA-DIRECT digital pretest information with standard-of-care pretest one-to-one genetic counselling

#### Randomisation

Using the on-line Sealed Envelope randomisation list generator, ahead of the study, we randomised study IDs 1:1 to receive pretest information digitally via the BRCA-DIRECT platform (fully digital pathway) or via telephone consultation with a genetics professional (partially digital pathway).^34^

#### Outcomes used for comparative evaluation of pretest information

1. Reported satisfaction and perceived convenience with the method in which they received the pretest information were compared using 5-point Likert scales.
2. Knowledge relating to BRCA-testing, assessed at baseline and 7 days after genetic test consent using a questionnaire comprising 14 ‘true’ or ‘false’ statements. Average (mean) overall scores and percentage of correct responses to individual questions were compared between the two arms at the two time points.
3. Patient anxiety, measured at baseline (T0), 7 days after BRCA-test consent (T1) and 7 (T2) and 28 days (T3) after receiving their results, using the Spielberger State-Trait Anxiety Inventory for Adults (STAI).^35^ The Intolerance of Uncertainty Scale (IUS) was also administered at baseline.^36^

### Study progression criteria

We established five progression criterion to support continuation from the pilot to a full powered study of 1000 patients, evaluating recruitment, retention, questionnaire completion, patient satisfaction with the digital intervention and change in knowledge from T0 to T1 in both the fully and partially digital arms (online supplemental table 1).

## Results

### Study population characteristics

**Figure 2:**
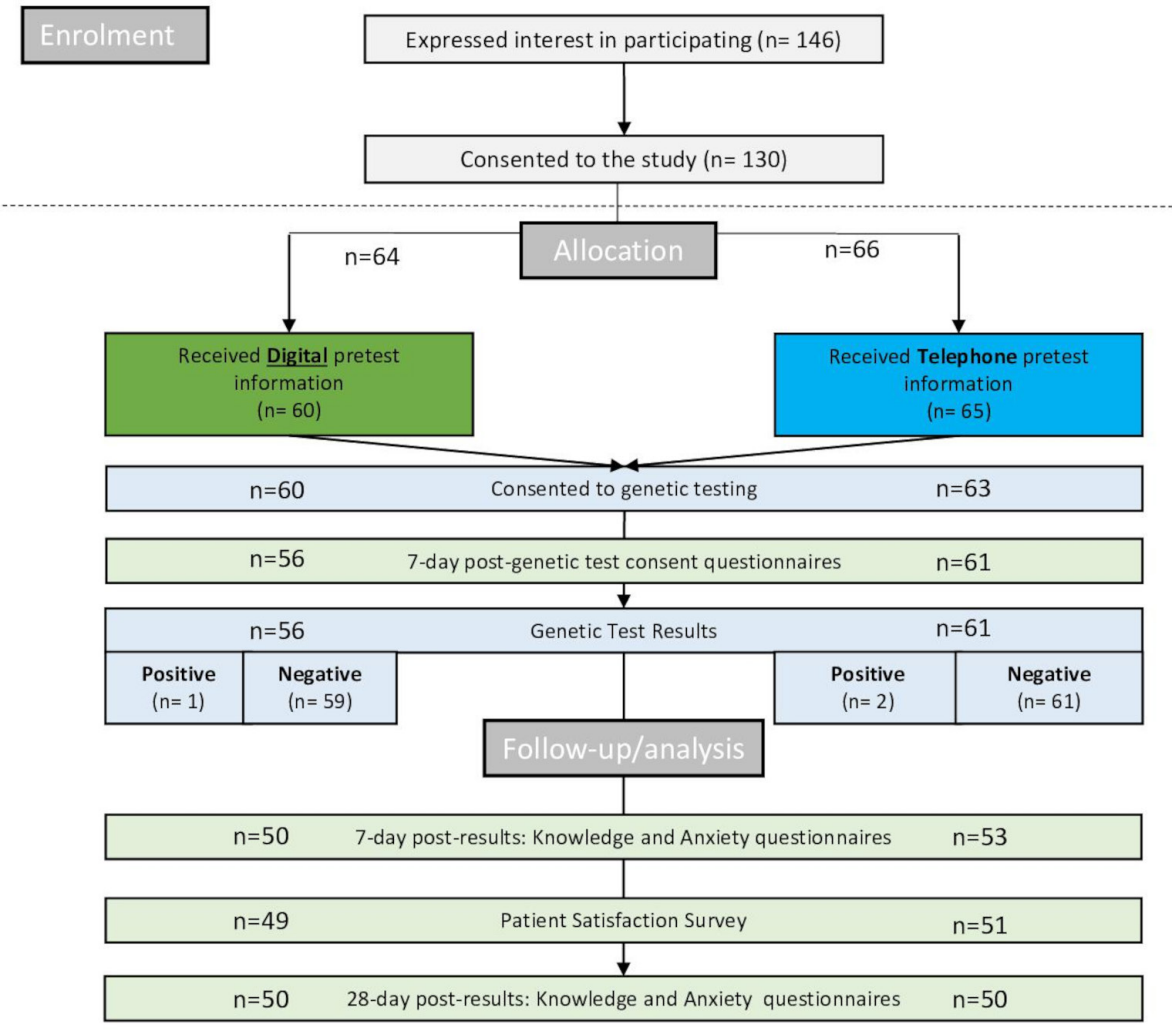
Consolidated Standards of Reporting Trials flow chart detailing patient progression through the BRCA-DIRECT pilot study, including number of patients included in analysis at each stage, separated by pretest information randomisation allocation following enrolment. Light green: study-specific outcome measures. Dark green: digital pretest information (fully digital arm). Dark blue: telephone pretest information (partially digital arm). Light blue: standard pathway procedures. See online supplemental figure 3 for more detail on patient progression and reasons for withdrawal or exclusions at each stage.

Recruitment to the BRCA-DIRECT internal pilot took place between 5 July 2021 and 10 October 2021 (97 days). During this time, 146 women with breast cancer expressed positive interest in participating, with 130 (89.0%) returning study consent forms and samples (figure 2; online supplemental figure 3).

Of the 130 women who consented to the study, 52.3% of patients were newly diagnosed and presurgical, 28.5% postsurgical under active treatment and the remainder under follow-up (6.9%) or metastatic (12.3%). Patients ranged from 33 to 87 years of age, with a mean age of 59 years old. See further demographics in table 1.

**Table 1:** BRCA-DIRECT pilot study patient demographics

Demographic	Groups	All	Pretest information allocation	
			Digital	Telephone
		N (%)	N (%)	N (%)
Age (years) (n=129)	18–30	0 (0.0)	0 (0.0)	0 (0.0)
	31–40	4 (3.1)	2 (2.9)	2 (3.4)
	41–50	27 (20.9)	13 (18.8)	14 (23.7)
	51–60	47 (36.4)	19 (27.5)	28 (47.5)
	61–70	25 (19.4)	15 (21.7)	10 (16.9)
	71–80	21 (16.3)	15 (21.7)	5 (8.5)
	81+	5 (3.9)	5 (7.2)	0 (0.0)
Ethnicity (n=128)	Asian or Asian British	8 (6.3)	5 (7.9)	3 (4.6)
	Black African, Caribbean or black British	3 (2.3)	0 (0.0)	3 (4.6)
	Mixed or multiple ethnic groups	7 (5.5)	3 (4.8)	4 (6.2)
	Other ethnic group	2 (1.6)	1 (1.6)	1 (1.5)
	Prefer not to say	2 (1.6)	2 (3.2)	0 (0.0)
	White	106 (82.8)	52 (82.5)	54 (83.1)
Highest education (n=128)	Higher degree level	31 (24.2)	13 (20.6)	18 (27.7)
	Degree level	39 (30.5)	21 (33.3)	18 (27.7)
	NVQ or equivalent	16 (12.5)	7 (11.1)	9 (13.8)
	A-levels	11 (8.6)	6 (9.5)	5 (7.7)
	GCSE or equivalent	23 (18.0)	12 (19.0)	11 (16.9)
	No qualification	4 (3.1)	2 (3.2)	2 (3.1)
	Prefer not to say	4 (3.1)	2 (3.2)	2 (3.1)
Marriage status (n=127)	Married or partnered	82 (64.6)	41 (66.1)	41 (63.1)
	Widowed	11 (8.7)	5 (8.1)	6 (9.2)
	Single	12 (9.4)	7 (11.3)	5 (7.7)
	Divorced	19 (15.0)	7 (11.3)	12 (18.5)
	Prefer not to say	3 (2.4)	2 (3.2)	1 (1.5)
Employment status (n=127)	Full time	47 (37.0)	20 (32.3)	27 (41.5)
	Part time	28 (22.0)	13 (21.0)	15 (23.1)
	Unemployed	11 (8.7)	6 (9.7)	5 (7.7)
	Retired	39 (30.7)	23 (37.1)	16 (24.6)
	Prefer not to say	2 (1.6)	0 (0.0)	2 (3.1)
Treatment stage (n=130)	New patient, presurgical	45 (34.6)	23 (35.9)	22 (33.3)
	New patient, presurgical, neoadjuvant chemo	23 (17.7)	8 (12.5)	15 (22.7)
	New patient, postsurgical, adjuvant therapy underway	37 (28.5)	19 (29.7)	18 (27.3)
	Under follow-up, disease-free, maintenance treatment only	9 (6.9)	4 (6.3)	5 (7.6)
	Metastatic	16 (12.3)	10 (15.6)	6 (9.1)

GCSE, General Certificate of Secondary Education; NVQ, National Vocational Qualification.

### Evaluation of the BRCA-DIRECT digital pathway

#### Uptake of BRCA-testing

Five (5/130) women withdrew from the study prior to receiving their pretest information. Of those who received pretest information, 123/125 (98.4%) consented to BRCA-testing; the two who withdrew were from the telephone (partially digital) arm (figure 2, online supplemental figure 3). See online supplemental table 2 for detail on withdrawals.

#### Hotline usage

The BRCA-DIRECT hotline was used overall by 24.7% of patients. Five ‘genetics specialist’ calls were made by patients (3.8% of all patients), seeking more information about what the results meant for the individual and their family members. Additional calls (63) were ‘administrative’ calls from 31 patients, with the majority relating to timing of results (n=35) and technical aspects of accessing the platform (n=18) (table 2).

**Table 2:** BRCA-DIRECT telephone hotline usage

Hotline calls recorded (n)				68
Average (mean) call length (min)				4.5
Patients using the hotline (n)				36
Proportion of participants using the hotline (%)				24.7
**Study stage of hotline call**			**N**	**%**
Prior to study consent			2	2.9
After study consent, up to receiving pretest information			22	32.4
After receiving pretest information, before completing genetic test consent			0	0.0
Awaiting results			37	54.4
After results/follow-up			7	10.3
**Type of call**			**N**	**%**
Genetics specialist		Pretest information	0	0.0
		Results	5	7.9
Administrative	Technical support	Sample provision	3	4.8
		Platform access or digital elements	15	23.8
	Process queries	Pathway/Study-specific	5	7.9
		Timing of results	35	55.6

#### Results turnaround

Samples were sequenced for all patients who completed their genetic test consent. Of the 123 results, there were 3 pathogenic variants, 0 likely pathogenic variants, 0 ‘hot’ VUS (5 evidence points) and 120 negative results. The overall median (IQR) time for testing of samples (from genetic-test consent to availability of results) was 27.6 (22.4–33.5) days, with similar turnaround times for patients in the fully digital (26.0 (20.6–33.2) days) and partially digital arms (28.6 (22.6–34.5) days) (online supplemental table 3).

#### Patient satisfaction with the pathway

**Figure 3:**
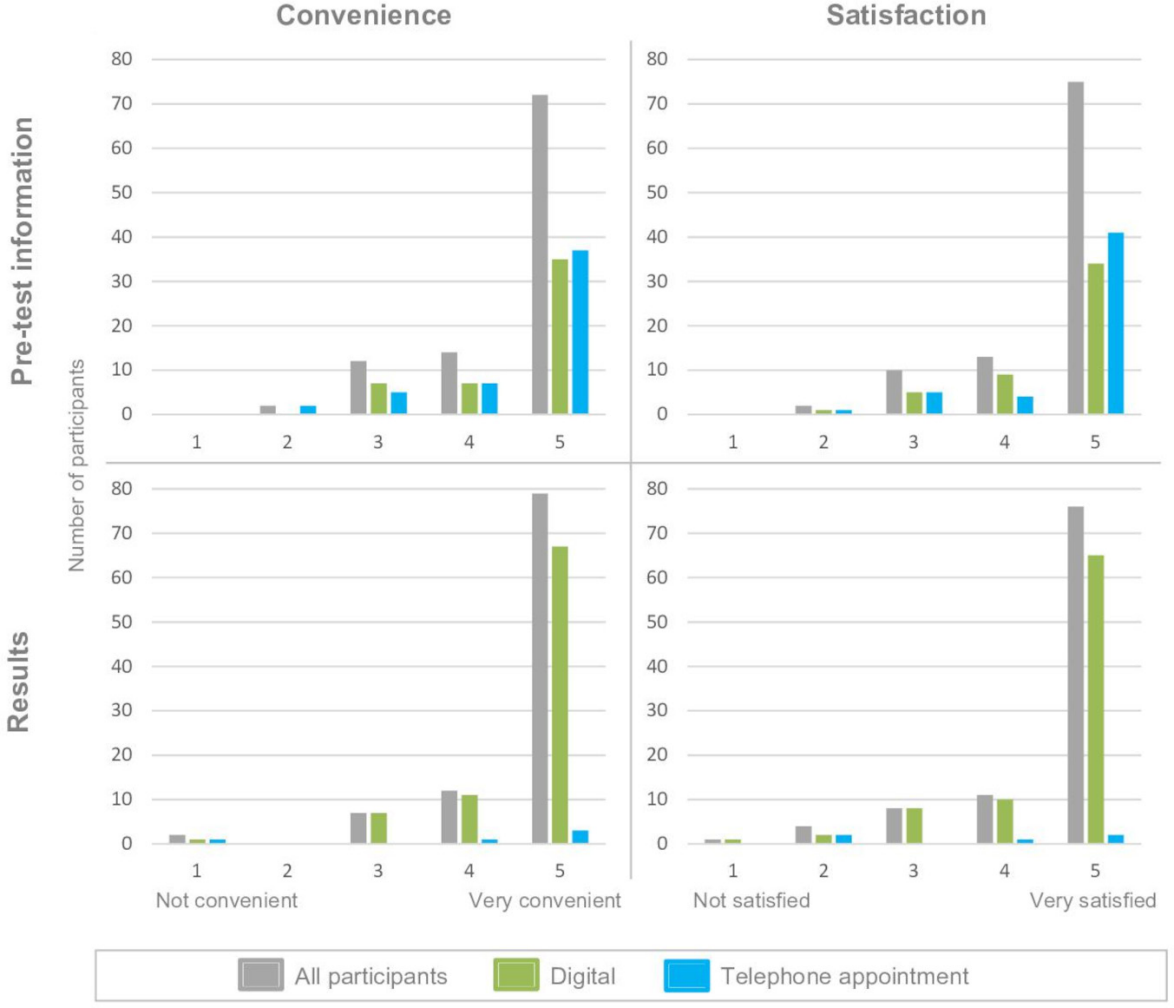
Patient-reported satisfaction and convenience. Patient Satisfaction Survey (PSS) completed by 100/130. Patient-reported convenience (1 not convenient–5 very convenient) and satisfaction (1 not satisfied–5 very satisfied). Digital (green) or telephone appointment (blue) delivery of pretest information (as per 1:1 randomisation) or results (as per random pre-allocation to telephone appointment for 2.5% of patients, plus for all patients receiving a variant of uncertain significance (VUS) or positive (pathogenic) result regardless of pre-allocation).

Patient-reported satisfaction and perceived convenience for (a) pretest information delivery and (b) delivery of results was overall high with 86% of responses being ≥4 (5=most convenient/satisfied); see figure 3 for comparison between digital and telephone pretest information. Seven per cent of patients reported seeking assistance with accessing the BRCA-DIRECT platform from clinical/study staff (2.0% (2/100)) or friends/family members (5.0% (5/100)), and 13% sought assistance with providing a saliva sample, 12.0% (12/100) from clinical/study staff and 1.0% (1/100) from friends/family members (online supplemental table 4).

Patients accessed the BRCA-DIRECT digital platform by a smartphone alone (45.0%), desktop computer/laptop alone (24.0%), tablet alone (8.0%) or from some combination of devices (23.0%) (online supplemental table 4). Patient interviews revealed that reminder notifications were useful (all scored either 4 or 5, with 5 being very useful and 1 being not useful). Of those who received both SMS and email reminders, there was an equal balance preferring SMS compared with email notifications, with patients noting that SMS notifications acted ‘as a reminder’ and the emails enabled an easy link to complete questionnaires via a computer or laptop.

#### Healthcare professional satisfaction with the pathway

Eleven HCPs responded to the survey (18.2% clinical nurses; 18.2% consultant breast oncologists; 36.4% consultant breast surgeons; 27.3% other). The majority agreed (to varying extents) that all aspects of the BRCA-DIRECT digital pathway were equivalent (or superior) to standard-of-care, with the exception of end-to-end time-to-results (27.3% disagreed, 45.5% agreed, 27.3% neither agreed nor disagreed) (online supplemental table 5). Overall, 72.7% perceived the benefits of the pathway to outweigh the challenges of the pathway (online supplemental table 6) and 80.0% believed that the BRCA-DIRECT pathway is ready to be implemented in the NHS.

### Randomised comparison of delivery of pretest information

Sixty patients, out of 125 (48.0%), were randomised to receive digital pretest information (fully digital pathway) and 65/125 (52.0%) were randomised to receive pretest information via telephone consultation with a genetics professional (partially digital pathway) (see figure 2).

Patient-reported satisfaction and convenience of delivery of pretest information were similar in both arms. In the fully digital arm, 85.7% of patients scored ≥4 for convenience and 87.8% scored ≥4 for satisfaction, compared with 86.3% and 88.2%, respectively in the telephone arm (figure 3). The amount of information and complexity of information were also considered to be ‘about right’ in the digital arm (89.8% and 91.8%), with figures being similar in the telephone arm (94.1% and 98.0%) (online supplemental table 7).

Following receipt of the pretest information, mean knowledge scores increased from 5.2/14 (SD 3.3) at baseline to 8.6/14 (SD 3.5) (online supplemental tables 8 and 9). The observed trend was similar between the digital (4.7/14 (SD 3.1) and 7.3/14 (SD 3.7)) and telephone arm (5.6/14 (SD 3.4) and 9.9/14 (SD 2.7)), as was the proportion of correct responses to individual questions in both arms (see online supplemental figure 5).

Mean (SD) anxiety scores decreased from the pretest baseline (T0) through to the ‘7 days post results’ time point (T2) in both the digital arm (45.1 (SD 13.6) at T0 and 37.3 (SD 12.9) at T2) and telephone arm (44.0 (SD 13.4) at T0, and 37.5 (SD 13.7) at T2). In both arms, results were similar at 7 days and 28 days postreceipt of results (online supplemental table 10). Baseline trait anxiety scores and IUS were similar in patients between the two arms (online supplemental table 10).

Safety reporting was conducted in line with the study protocol and ethics approvals. No serious adverse events relating to the fully or partially digital pathways were recorded during the BRCA-DIRECT pilot.

### Progression criteria

All progression criteria established to support continuation of the study were met or exceeded (online supplemental table 11).

## Discussion

We have presented data from our pilot of the BRCA-DIRECT pathway in the first 130 unselected patients with breast cancer from mainstream oncology services in 3 NHS hospitals, of whom half had the fully digital pathway (digital pretest information) and half had the partially digital pathway (telephone consultation pretest information). Considering the fully digital BRCA-direct pathway: uptake of BRCA-testing was high (60/64, 93.8%, with all withdrawals being prior to pretest information), as were ratings for perceived convenience and satisfaction for how they received pretest information and results (42/49 (87.8%) scoring as 4–5/5). Preliminary data regarding delivery of pretest information showed similar patient knowledge score, anxiety or satisfaction scores for the digital delivery and telephone genetic counselling.

As expected from general internet-access patterns, the BRCA-DIRECT platform was mainly accessed via a smartphone. However, a mixture of devices were used, demonstrating the importance of optimising the digital platform across different devices. Usability of the BRCA-DIRECT digital pathway was demonstrated to be high, with low numbers of patients requiring support, as indicated by both patient feedback (5.0% stated they sought technical support from another person) and analysis of calls placed to the hotline (23.8% of patients made a hotline call for administrative support regarding the platform). Notably, only 3.8% of patients accessed the hotline for expert genetics support, with all of these relating to results rather than pretest information. Similar hotline usage patterns have been reported by Gaba *et al* 37 in unselected population-based personalised ovarian cancer risk assessment.

The majority of patient hotline calls placed were administrative and related to availability of results (35/68 (55.9%)). The time-to-results (median (IQR) time from receipt of sample (and study consent) to return of results) was 38.4 days (31.3–48.8) and testing turnaround time (time from genetic test consent to results available) was 27.6 days (22.4–33.5), reflecting the communicated turnaround time estimation of 3–4 weeks. However, the upper quartile of testing turnaround times experienced significant delays, reflective of the impact over this period of COVID-19-related supply chain issues for reagents and staffing shortages. Permissive estimates of turnaround time and provision on the digital platform of clear and accurate timeframes for turnaround of results is clearly critical for successful implementation and scaling of a fully digital pathway.

Five patients, out of 123, either failed to confirm that their results were received digitally or failed to book an appointment following notification of the results being available. Possible explanations included return of results coinciding with in-patient or treatment activity or patient demise. An alert was placed to the respective oncology professional, ensuring diversion to clinician-directed return of results. Such deviations illustrate the importance of integration of the digital pathway within oncology care delivery, ensuring both clinician awareness regarding patient progression with genetic testing and that results have been returned.

### Limitations of study

The randomisation pertained to just delivery of pretest information, not the full pathway. This allowed us to perform a direct comparison of groups between which only delivery of pretest information differed. However, for those in the telephone (partially digital) arm, digital appointment bookings were likely more accessible, rapid and flexible than a standard NHS clinical service. In that regard, a study of randomisation between an NHS standard-of-care pathway versus a BRCA-DIRECT fully digital pathway would be informative; this was not feasible as NHS clinical appointments could not be allocated to patients not eligible for NHS testing. HCP feedback indicated areas where the pathway was equivalent (or superior) to standard care, however, number of responses was limited.

Accessibility on account of both digital literacy/access and language was identified by HCPs as one of the main challenges/shortcomings to the BRCA-DIRECT digital pathway. Ability and willingness to access a digital platform was one of the criteria for eligibility. Thus, our randomised comparison only pertained to this restricted subset of patients with breast cancer, although that we explicitly allowed study participation for those using the device/credentials of a trusted nominee. We sought to capture the reasons for patients declining participation in the study, but were limited to only those willing to offer such a response and could not collect detailed demographics on this group.

The eligibility criterion requiring patients to have a good comprehension of English was established to protect the safety and integrity of patients, aiming to ensure comprehension of the digital pretest information and subsequent informed consent to genetic testing, as well as enabling patients to proceed through the digital tasks by responding to notifications. Developing and translating the digital platform, notifications and pretest information was beyond the limitations of this study but should be considered in any broader rollout.

Additionally, it was challenging to ensure that in their feedback, patients were accurately differentiating the core BRCA-DIRECT pathway (saliva sample, core baseline information, digital pretest information, test consent, return of results) from the elements of the process relating to the evaluative study (study consent, extra baseline info, knowledge scores and STAI): from patient interviews and patient-reported method of pretest information/results delivery, there was evidence of the two being conflated.

### Future developments

The outcomes of the established progression criteria supported study continuation, with all criteria met or exceeded. Based on the findings and feedback from the semi-qualitative interviews, minor adaptations have been made to the digital pretest information, knowledge survey and patient reminders, largely to improve consistency of language and clarity of instructions. Based on the turnaround times, hotline usage and satisfaction surveys, adaptations to the sample-laboratory pathway have been implemented, with continued attention to the feasibility of returning results sufficiently rapidly to reliably inform surgical decision-making in those proceeding to surgery without neoadjuvant chemotherapy. With these adaptations, we shall progress to the full study of 1000 women, for a highly powered comparison of the two groups regarding pretest information, detailed operational outcomes, in-depth qualitative assessment of patient-centred outcomes and operational economic analyses.

Thus, in summary, these data provide no evidence thus far of ‘harms’ regarding increased anxiety or reduction in knowledge relating to the ‘fully digital’ BRCA-DIRECT pathway. These data offer a preliminary demonstration of feasibility and acceptability of a fully digital pathway for BRCA-testing, which is more rapid and patient-centred than the conventional pathway, while maintaining the tight integration into NHS clinical and lab infrastructures along with access as required by genetics professionals. There is potential for an NHS-integrated, patient-centred, clinician-light, digital pathways opportunity such as this to enable substantial expansion of germline genetics for BRCA-testing as well as for other use-cases in oncology and other areas of medicine.

## Data Availability

Data are available on reasonable request. Data from this study are not publicly available due to inclusion of special category and sensitive data. Data access requests can be made to the corresponding author for consideration.
