# Supplementary material for: A digital pathway for genetic testing in UK NHS patients with cancer: BRCA-DIRECT randomised study internal pilot
Source: J Med Genet. 2022 Jul 22;59(12):1179–88. doi: 10.1136/jmg-2022-108655 (PMC9691828; doi:10.1136/jmg-2022-108655)
Supplement: Supplementary data [file jmg-2022-108655supp001.pdf]

A digital pathway for genetic testing in UK NHS cancer patients: BRCA-DIRECT randomised study internal pilot

B. Torr[1], C. Jones[2], S. Choi[1], S. Allen[1], G. Kavanaugh[1], M. Hamill[1], A. Garrett[1], S. MacMahon[3], L. Loong[1], A. Reay[3], L. Yuan[3], M. Valganon Petrizan[3], K. Monson[4], N. Perry[2], L. Fallowfield[4], V. Jenkins[4], R. Gold[5], A. Taylor[6], R. Gabe[7], J. Wiggins[8], A. Lucassen[9,10], R. Manchanda[7,11,12], A. Gandhi[13], A. George[1,8], M. Hubank[3], Z. Kemp[1,8], D.G. Evans[14, 15], S. Bremner[2] and C. Turnbull[1, 8]

[1] Division of genetics and Epidemiology, The Institute of Cancer Research, London, UK
[2] Brighton and Sussex Clinical Trials Unit, University of Sussex, Sussex, UK
[3] Centre for Molecular Pathology, The Institute of Cancer Research, London, UK
[4] Sussex Health Outcomes Research and Education in Cancer (SHORE-C), Brighton and Sussex Medical School, University of Sussex, Sussex, UK
[5] Patient Representative, BRCA Journey, Leeds, UK
[6] Department of Medical Genetics, Cambridge University Hospitals NHS Foundation Trust, Cambridge, UK
[7] Wolfson Institute of Population Health, Queen Mary's University of London, London, UK
[8] Cancer Genetics Unit, Royal Marsden NHS Foundation Trust, London, UK
[9] Nuffield Department of Medicine, University of Oxford, Oxford, UK
[10] Clinical Ethics and Law Unit, Faculty of Medicine, University of Southampton, Southampton, UK
[11] Department of Gynaecological Oncology, Barts Health NHS Trust, London, UK
[12] Department of Health Services Research, Faculty of Public Health & Policy, London School of Hygiene and Tropical Medicine, London, UK
[13] Nightingale and Genesis Breast Cancer Centre, University Hospital of South Manchester, Manchester, UK
[14] Division of Evolution and Genomic Sciences, The University of Manchester, Manchester, UK
[15] Mangen Centre, Manchester University NHS Foundation Trust, Manchester, UK

Corresponding author: Professor Clare Turnbull, Division of Genetics and Epidemiology, Institute of Cancer Research, Sutton SM2 5NG, UK; clare.turnbull@icr.ac.uk

Supplementary Methods

Torr B, *et al. J Med Genet* 2022; 59:1179–1188. doi: 10.1136/jmg-2022-108655

BRCA-DIRECT PATHWAY

Patient DNA sampling

Patient saliva samples were collected using Isohelix Genefix (GFX) Saliva DNA/RNA Collection and Stabilization kits. Patients were provided with instructions on how to collect their sample independently in clinic or at home with postal return to minimise aerosol generating procedures during the COVID19 pandemic. A blood sample pathway was also made available for patients already having clinical/routine bloods taken.

Laboratory analysis

Genetic analysis was undertaken at The Centre for Molecular Pathology (Royal Marsden NHS Foundation Trust and Institute of Cancer Research, London, UK) accredited to International Organization for Standardization (ISO) 15189:2012 (UKAS laboratory accreditation: 20653 Medical Single (ukas.com)).

The exonic regions of BRCA1 (ENST00000357654), BRCA2 (ENST00000380152) and PALB2 ((ENST00000261584)) and flanking intronic regions of 20bps were amplified using a bespoke Qiagen Qiaseq targeted DNA panel and sequenced using Illumina technologies via MiSeq/Nextseq platforms. Alignment and calling of small variants and copy number variants was undertaken using a customised pipeline comprising an in-house bioinformatics pipeline validated against a panel of known BRCA1 and BRCA2 variants*. Multiplex Ligation-dependent Probe Amplification (MLPA) (MRC-Holland kit) was performed to evaluate suspected copy number variants (CNVs).

Variants were classified according to the ACMG-AMP variant classification framework, using the specifications detailed by UK-ACGS (UK Association of Clinical Genomic

Torr B, *et al. J Med Genet* 2022; 59:1179–1188. doi: 10.1136/jmg-2022-108655

Scientists) and CanVIG-UK (Cancer variant interpretation Group UK)[1], [2], [3]. As per recommendations of UK-ACGS (UK Association of Clinical Genomic Scientists), the classification was presented using evidence (exponent) points and only variants classified as pathogenic, likely pathogenic or hot variants of uncertain significant (hot VUS) (4/5 evidence points) were included on the laboratory report. [3], [2], [4]

BRCA-DIRECT Telephone Hotline

A telephone hotline was available for patients 9am-to-5pm, Monday-to-Friday (excluding public holidays). The hotline was staffed by genetics professionals (genetic counsellor, onco-genetics specialist nurse or clinical geneticist) to support with calls requiring specialist knowledge about genetic testing and/or the results. Research administrators were also available to support with technical or administrative calls only.

Details of the telephone hotline were widely visible within the BRCA-DIRECT digital platform and across study materials available to patients from initial approach about BRCA-DIRECT. All calls to the hotline were logged via the BRCA-DIRECT platform with the following details recorded: date of call, length of call, call handler and free-hand notes.

BRCA-DIRECT Digital Platform

The BRCA-DIRECT digital platform consisted of a custom-built database and website housed on isolated, secure servers at the Institute of Cancer Research and integrated with email and SMS messaging systems.

Data security and information governance provisions were incorporated into the design and build of the BRCA-DIRECT platform for handling special category and patient identifiable data and ensuring compliance with relevant governing principles, including General Data Protection Regulation (GDPR).

Independent and moderated user-testing of the platform functionality and content was conducted with clinical genetics specialists, researchers, and patients and the platform optimised for usability and functionality across different devices. The resulting features of the BRCA-DIRECT digital platform are outlined below.

Role-specific administrative access

Administrative functionality of the platform included: (i) adding/amending patient profiles, (ii) management of patient workflow, (iii) tracking patient samples, (iv) logging telephone appointments and hotline calls, (v) management of results, and (vi) generating letters. Administrative user access was specified by role (laboratory user, hospital site-specific clinical user, or genetic counsellor users) to ensure minimal need-to-know access to specific patient data.

Patient user access

Patient user profiles were created by administrative users upon receipt of an expression of interest (EOI) or study consent form, enabling participants to register for a personalised account and receive automated reminders via email and/or SMS to complete tasks within specified conditions

Patient workflow

The patient workflow was structured into ten steps, which comprised both those elements core to the BRCA-DIRECT pathway (eg collection of critical baseline information, pre-test information, test consent and test results), and elements related to the evaluative study (questionnaires and surveys)(see supplementary figure 2).

Upon login, the patient's position in the workflow was reflected to them. Following completion of each step, the next step would be presented to the patient via a pop-up

Torr B, *et al. J Med Genet* 2022; 59:1179–1188. doi: 10.1136/jmg-2022-108655

explanation. Progression and access to specific steps was automated based on (i) timing, (ii) confirmed completion of a previous step, or (iii) administrative update.

BRCA-DIRECT digital pre-test information

Digital pre-test information was developed to be of equivalent content and depth to a standard genetic counselling appointment. The materials were developed by the study team and evolved for language, content and presentation via iterative consultation with our clinical and patient user-groups. The final package comprised 21 static screens of written information and schematics. These included 13 core screens covering topics such as genetic inheritance, cancer risk and the process of BRCA-testing and seven screens entitled “more detailed information”, which addressed insurance, options for risk reduction and oncogenesis (Appendix 1).

After viewing all screens, patients were advised to contact the genetic specialist hotline if they required additional support with understanding the information and were required to confirm they had received the pre-test information before proceeding to the digital genetic test consent form.

Digital genetic test consent

The genetic test consent form was designed to reflect directly the contents of the ‘Record of Discussion Regarding Genomic Testing’ used in the UK NHS Genomic Medicine Service, and evolved following consultation with our clinical and patient user-groups.

The digital consent form required participants to mark that they had read and confirmed each statement, followed by typing a digital signature and confirming the date. A confirmatory pop-up notice was required to be acknowledged by the patient upon submission on the form. After confirmation, a PDF copy of the consent form was

Torr B, *et al. J Med Genet* 2022; 59:1179–1188. doi: 10.1136/jmg-2022-108655

made available for the patient to download. A copy was also sent to the patients GP and added to the hospital medical record.

Return of BRCA-test results

Availability of results was communicated to the patient from the BRCA-DIRECT platform via SMS and/or email and, upon login to the platform, the patient received their result either digitally via the BRCA-DIRECT platform or was invited to book a 1:1 telephone consultation with a genetics professional (half-day telephone slots (9am-12pm or 1pm-4pm) available from the following day).

Participants were pre-allocated to receive results digitally (97.5%) or via telephone consultation (2.5%) using the on-line Sealed Envelope™ randomisation list generator.[5] All those with a negative result received their result according to the pre-allocated randomisation. Participants with a reported variant (hot VUS, Likely Pathogenic, Pathogenic), predicted maximum 5% of participants, received their result via telephone consultation, regardless of pre-allocated randomisation.

Formal Communication of BRCA-test results and patient follow-up

A summary letter was automatically generated according to the result of negative (no variants reported), positive (Pathogenic, Likely Pathogenic variant reported) or VUS. The letter was auto-populated with the patients' clinical diagnosis, self-reported family history, test outcomes and accordant clinical recommendations (including screening recommendations predicated on family/personal cancer history for those with negative results) and was sent with the laboratory report to the patient, GP and hospital clinical team. For patients in whom a VUS/Likely Pathogenic/Pathogenic variant is reported, a clinical genetics referral letter was also generated.

REFERENCES

1. Richards S, Aziz N, Bale S, Bick D, Das S, Gastier-Foster J, Grody WW, Hegde M, Lyon E, Spector E, Voelkerding K, Rehm HL. Standards and guidelines for the interpretation of sequence variants: a joint consensus recommendation of the American College of Medical Genetics and Genomics and the Association for Molecular Pathology. Genet Med 2015;**17**(5):405-24 doi: 10.1038/gim.2015.30[published Online First: Epub Date]|.
2. Garrett A, Durkie M, Callaway A, Burghel GJ, Robinson R, Drummond J, Torr B, Cubuk C, Berry IR, Wallace AJ, Ellard S, Eccles DM, Tischkowitz M, Hanson H, Turnbull C. Combining evidence for and against pathogenicity for variants in cancer susceptibility genes: CanVIG-UK consensus recommendations. J Med Genet 2020 doi: 10.1136/jmedgenet-2020-107248[published Online First: Epub Date]|.
3. Ellard S, Baple EL, Berry I, Forrester N, Turnbull C, Owens M, Eccles DM, Abbs S, Scott R, Deans Z, Lester T, Campbell J, Newman W, McMullan D. ACGS Best Practice Guidelines for Variant Classification 2020: Association for Clinical Genetics Science (ACGS), 2020.
4. Tavtigian SV, Harrison SM, Boucher KM, Biesecker LG. Fitting a naturally scaled point system to the ACMG/AMP variant classification guidelines. Hum Mutat 2020 doi: 10.1002/humu.24088[published Online First: Epub Date]|.
5. Sealed Envelope Ltd. Create a blocked randomisation list. Secondary Create a blocked randomisation list. 2021.
