# Supplementary material for: A digital pathway for genetic testing in UK NHS patients with cancer: BRCA-DIRECT randomised study internal pilot
Source: J Med Genet. 2022 Jul 22;59(12):1179–88. doi: 10.1136/jmg-2022-108655 (PMC9691828; doi:10.1136/jmg-2022-108655)
Supplement: Supplementary data [file jmg-2022-108655supp002.pdf]

A digital pathway for genetic testing in UK NHS cancer patients: BRCA-DIRECT randomised study internal pilot

B. Torr[1], C. Jones[2], S. Choi[1], S. Allen[1], G. Kavanaugh[1], M. Hamill[1], A. Garrett[1], S. MacMahon[3], L. Loong[1], A. Reay[3], L. Yuan[3], M. Valganon Petrizan[3], K. Monson[4], N. Perry[2], L. Fallowfield[4], V. Jenkins[4], R. Gold[5], A. Taylor[6], R. Gabe[7], J. Wiggins[8], A. Lucassen[9,10], R. Manchanda[7,11,12], A. Gandhi[13], A. George[1,8], M. Hubank[3], Z. Kemp[1,8], D.G. Evans[14, 15], S. Bremner[2] and C. Turnbull[1, 8]

[1] Division of genetics and Epidemiology, The Institute of Cancer Research
[2] Brighton and Sussex Clinical Trials Unit, University of Sussex
[3] Centre for Molecular Pathology, The Institute of Cancer Research
[4] SHORE-C, Brighton & Sussex Medical School, University of Sussex
[5] Patient Representative, BRCA Journey
[6] Department of Medical Genetics, Cambridge University Hospitals NHS Foundation Trust
[7] Wolfson Institute of Population Health, Queen Mary's University of London
[8] Cancer Genetics Unit, Royal Marsden NHS Foundation Trust
[9] Nuffield Department of Medicine, University of Oxford
[10] Clinical Ethics and Law Unit, Faculty of Medicine, University of Southampton
[11] Department of Gynaecological Oncology, Barts Health NHS Trust, London, UK
[12] Department of Health Services Research, Faculty of Public Health & Policy, London School of Hygiene & Tropical Medicine, London, UK
[13] Nightingale & Genesis Breast Cancer Centre, University Hospital of South Manchester
[14] Division of Evolution & Genomic Sciences, The University of Manchester
[15] Mangen Centre, Manchester University NHS Foundation Trust

Supplementary Tables and Figures

Supplementary figure 1: Overview of the BRCA-DIRECT digital pathway, encompassing comparison between delivery of pre-test information by digital format (fully digital pathway, green) or telephone appointment with a genetics professional (partially digital pathway, blue). 3

Supplementary figure 2: An overview of the BRCA-DIRECT digital platform patient (blue) and administrative (orange) functionality with supportive images from the online webpages.......... 4

Supplementary table 1: BRCA-DIRECT pilot study progression criteria.......... 5

Supplementary figure 3: CONSORT flowchart detailing patient progress, withdrawals at each stage and exclusions from analysis. Light green: study-specific outcome measures. Dark green: fully-digital pathway with randomisation allocation to digital pre-test information. Dark blue: comparator partially-pathway. Light blue: standard pathway procedures. 6

Supplementary table 2: Details of patient withdrawal following study consent 7

Supplementary table 3: Median number of days taken to progress through the BRCA-DIRECT pathway from point of study consent to delivery of results (time-to-results), separated by study stage (1-4) and pre-test information allocation arm (digital or telephone appointment). See supplementary figure 4 for an overview of pathway stages.......... 8

Torr B, *et al. J Med Genet* 2022; 59:1179–1188. doi: 10.1136/jmg-2022-108655

Supplementary figure 4: BRCA-DIRECT pathway stages. 1) Pre-test information delivery; 2) decision to proceed with genetic test; 3) testing of sample; 4) results delivery. 8

Supplementary table 4: Patient reported access and support requirements during involvement with the BRCA-DIRECT digital pathway. 9

Supplementary table 5: Healthcare professional reported satisfaction with elements of the BRCA-DIRECT digital pathway compared with standard care 9

Supplementary table 6: Healthcare professional reported benefits and challenges/short-falls of the BRCA-DIRECT digital pathway 10

Supplementary table 7: Patient reported feedback on the content of pre-test information delivered either digitally via the BRCA-DIRECT platform (fully-digital pathway) or via a telephone appointment with a genetics specialist nurse or counsellor (partially-digital pathway). 10

Supplementary table 8: Average (mean) knowledge scores out of a total of 14 points at baseline (T0) and 7-days post-genetic test consent (T1) in all patients, patients randomised to receive pre-test information digitally only (fully-digital pathway), and patients randomised to receive pre-test information via telephone consultation with a genetics professional only (partially-digital pathway). 11

Supplementary table 9: Knowledge score questions and number of patients answering individual knowledge questions correctly in the digital and telephone pre-test information arms at baseline (T0) and 7-days post-genetic test consent. 12

Supplementary figure 5: Knowledge test: Percentage of patients answering individual knowledge questions correctly and the overall average (mean) knowledge score for patients (% correct out of a total of 14) in the digital (green) and telephone (blue) pre-test information arms at baseline (T0) (lighter bars) and 7-days post-genetic test consent (T1) (darker bars). 13

Supplementary table 10: Mean Intolerance of Uncertainty, 'trait' anxiety (State Trait Anxiety Index (STAI) Y2) and 'state' anxiety (STAI Y1) scores of all patients and those randomised to receive digital pre-test information or telephone pre-test information only. Mean 'state' anxiety scores (STAI Y1) are captured at baseline (T0), 7-days post-genetic test consent (T1), 7-days post-genetic test results (T2), and 28-days post-genetic test results (T3) and exclude patients who completed >5 days after the time point. 14

Supplementary table 11: Progression criteria outcomes. 15

Supplementary figure 1: Overview of the BRCA-DIRECT digital pathway, encompassing comparison between delivery of pre-test information by digital format (fully digital pathway, green) or telephone appointment with a genetics professional (partially digital pathway, blue).

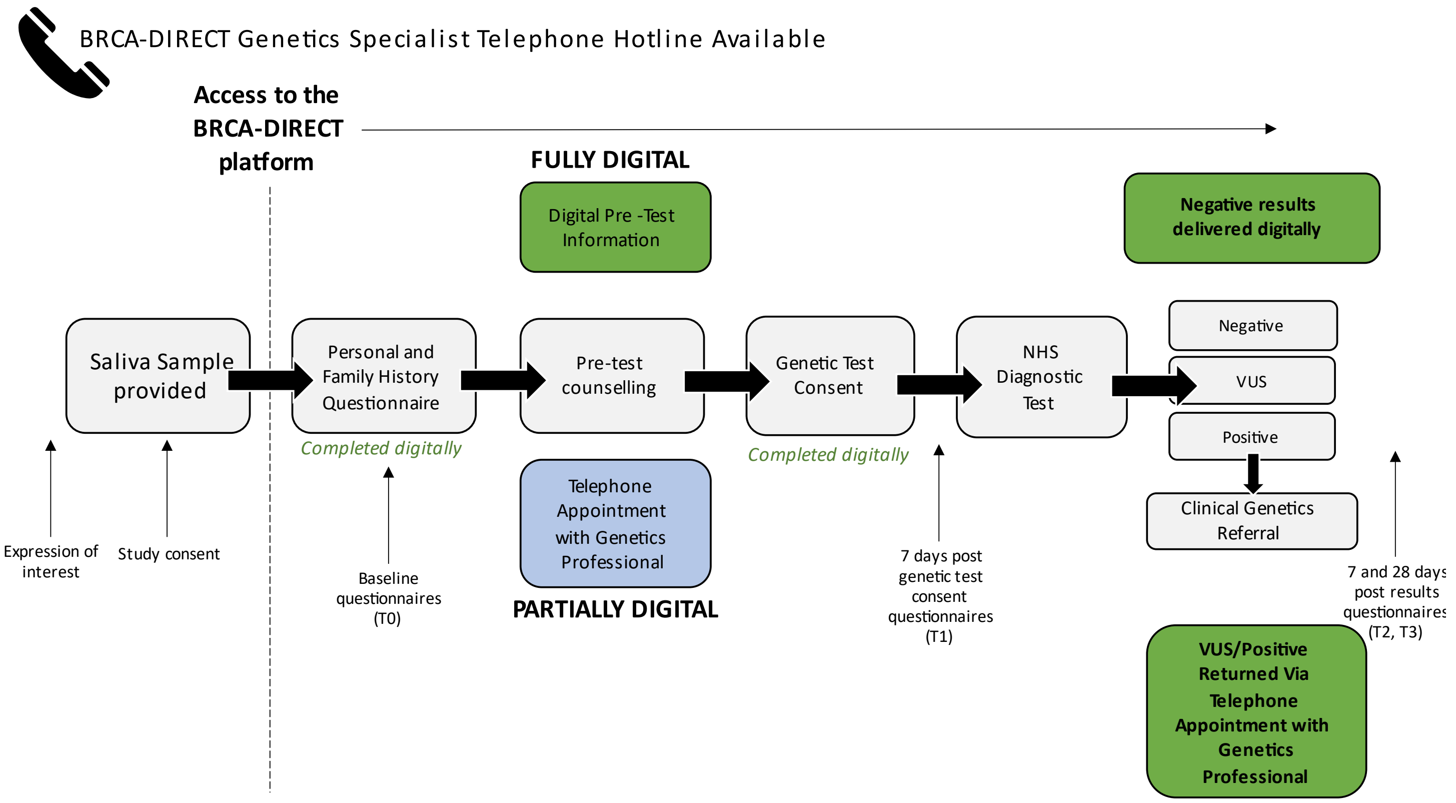

Torr B, *et al. J Med Genet* 2022; 59:1179–1188. doi: 10.1136/jmg-2022-108655

Supplementary figure 2: An overview of the BRCA-DIRECT digital platform patient (blue) and administrative (orange) functionality with supportive images from the online webpages.

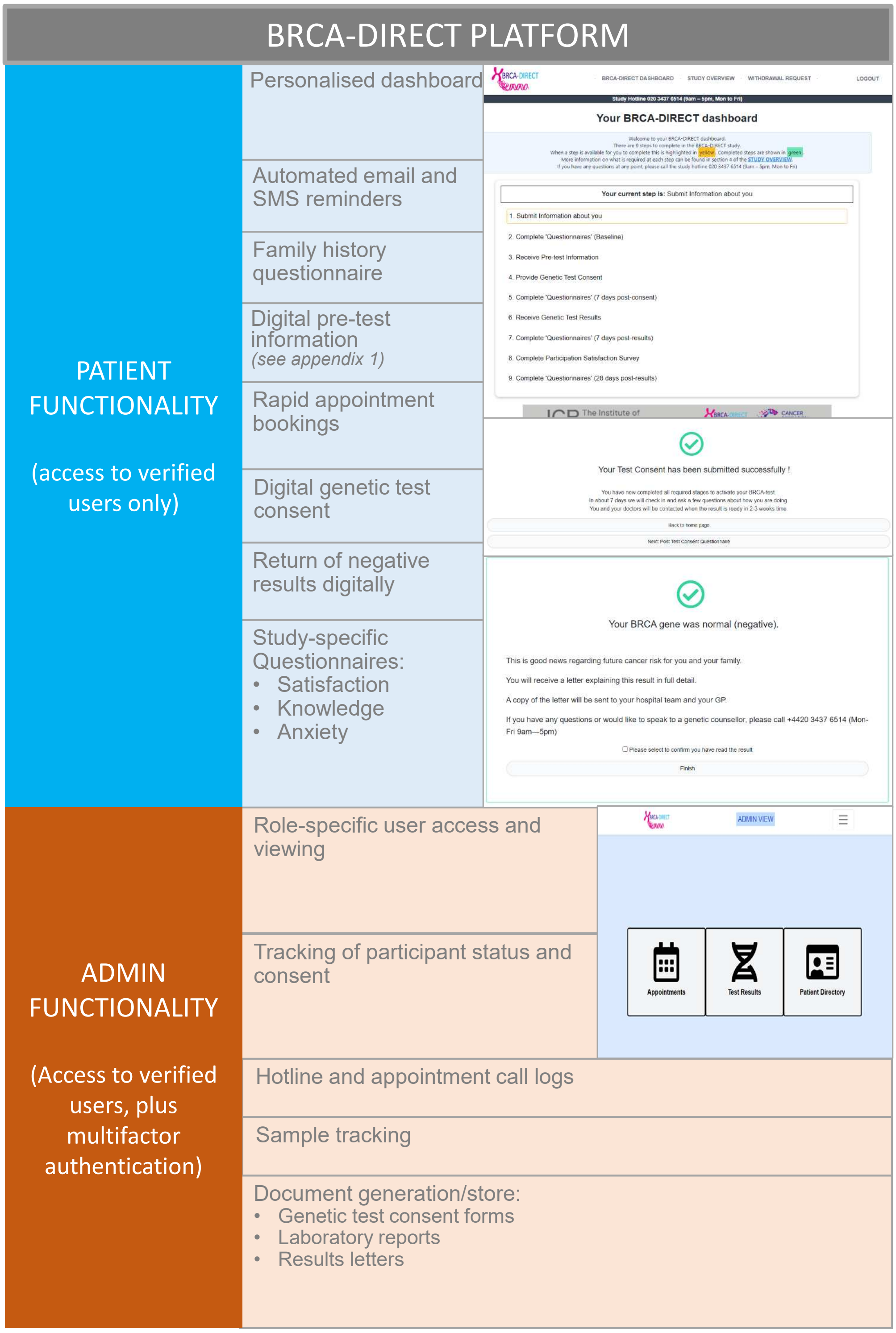

4

Supplementary table 1: BRCA-DIRECT pilot study progression criteria

**Progression Criterion**		
**1**	Recruitment rate	Recruitment rate during the pilot is >90% of what is required to meet the full study target (1000 patients recruited across two hospital trusts equivalent in case load).
**2**	Retention	Retention is >75% at final follow-up.
**3**	Questionnaire completion compliance	>80% of patients complete surveys within a window of 7 days of the expected deadline.
**4**	Satisfaction with the digital intervention	A satisfaction score of ≥3 in at least 80% of patients for questions 9a (How satisfied were you with the way you received your pre-test information?) and 9b (How satisfied were you with the way you received your results?) of the Patient Satisfaction Survey (PSS).
**5**	Change in knowledge following pre-test information	Average (mean) knowledge questionnaire score at T1 at least as high as the average at T0 in both arms.

Torr B, *et al. J Med Genet* 2022; 59:1179–1188. doi: 10.1136/jmg-2022-108655

Supplementary figure 3: CONSORT flowchart detailing patient progress, withdrawals at each stage and exclusions from analysis. Light green: study-specific outcome measures. Dark green: fully-digital pathway with randomisation allocation to digital pre-test information. Dark blue: comparator partially-pathway. Light blue: standard pathway procedures.

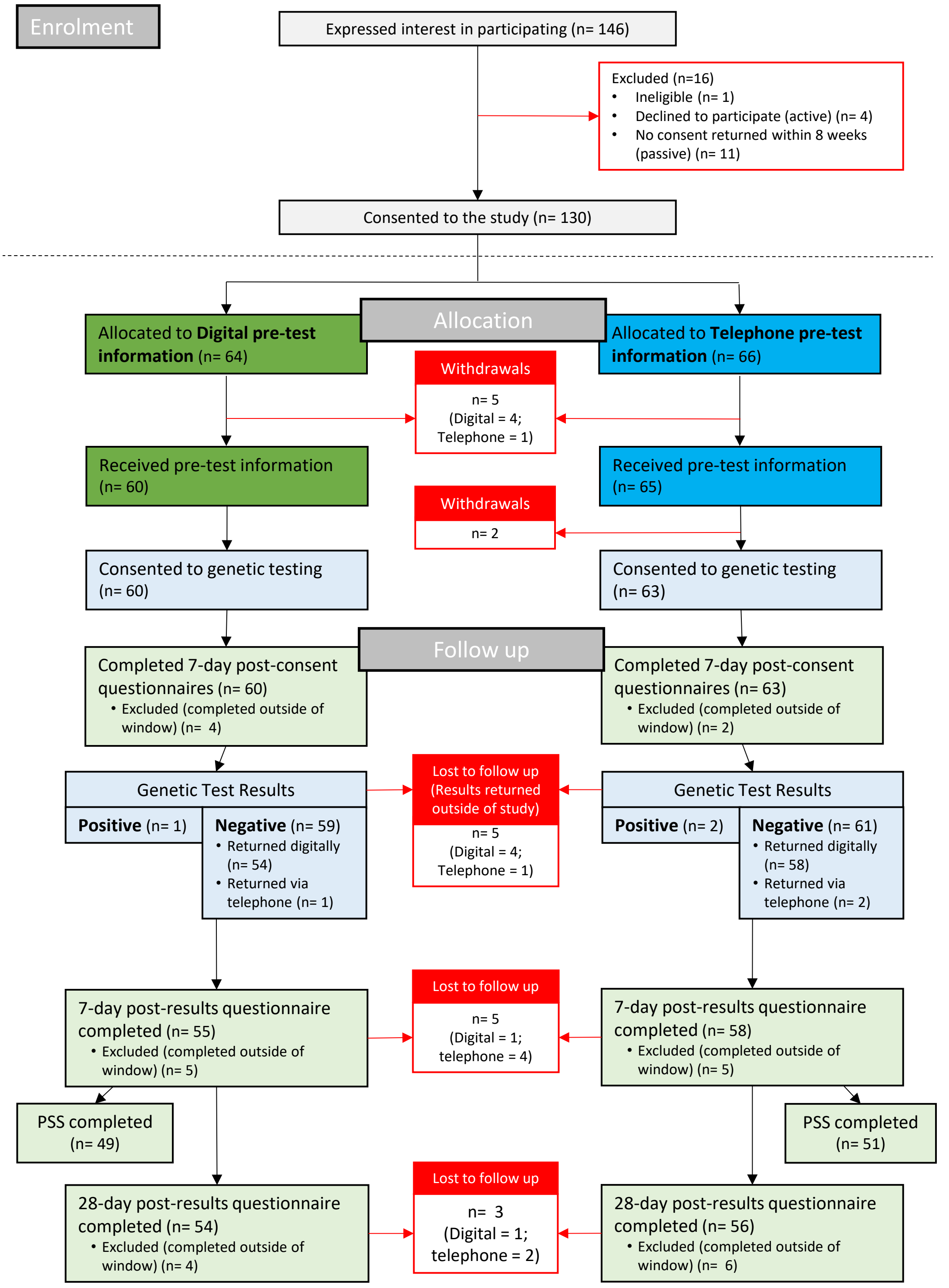

Torr B, *et al. J Med Genet* 2022; 59:1179–1188. doi: 10.1136/jmg-2022-108655

Supplementary table 2: Details of patient withdrawal following study consent

Withdrawal characteristics		All (*n*)	Digital (*n*)	Telephone (*n*)
	Total withdrawals*	20	10	10
Stage of withdrawal	Prior to creating a BRCA-DIRECT account	1	1	0
	Before receiving pre-test information	4	3	1
	After receiving pre-test information, Prior to genetic test consent	2	0	2
	Failed to receive results**	5	4	1
	Follow up (7/28-day follow up questionnaires)	8	2	6
Type of withdrawal	Active withdrawal (patient contacted study)	2	1	1
	Passive withdrawal (>6 weeks, no activity)	18	9	9
Reason for withdrawal	Too many digital steps to complete	1	1	0
	A lot going on, not the right time for study participation/genetic testing	3	0	3
	Failure to progress, reason unknown	16	9	7

**5/123 patients failed to confirm that they had received their results after viewing digitally. Possible explanations for non-receipt via the BRCA-DIRECT pathway included return of results coinciding with in-patient or treatment activity or patient decease. An alert was placed to the respective oncology professional, to ensure clinician-directed return of results.

Torr B, *et al. J Med Genet* 2022; 59:1179–1188. doi: 10.1136/jmg-2022-108655

Supplementary table 3: Median number of days taken to progress through the BRCA-DIRECT pathway from point of study consent to delivery of results (time-to-results), separated by study stage (1-4) and pre-test information allocation arm (digital or telephone appointment). See supplementary figure 4 for an overview of pathway stages.

Stage		All		Pre-test information allocation			
				Digital		Telephone	
		n	*Median Days (IQR)*	*n*	*Median Days (IQR)*	*n*	*Median Days (IQR)*
1	Pre-test information delivery	125	8.4 (5.5 - 12.6)	60	7.4 (4.3 - 13.2)	65	8.6 (6.6 - 12.6)
2	Decision to proceed with genetic test*	123	0.0 (0.0 - 0.0)	60	0.0 (0.0 - 0.0)	63	0.0 (0.0 - 0.0)
3	Testing of sample**	123	27.6 (22.4 - 33.5)	60	26.0 (20.6 - 33.2)	63	28.6 (22.6 - 34.5)
4	Results delivery	118	0.0 (0.0 - 1.4)	56	0.0 (0.0 - 0.3)	62	0.1 (0.0 - 3.8)
**Time-to-results**		**118**	**38.4 (31.3 - 48.8)**	**56**	**37.6 (28.7 - 45.6)**	**62**	**40.0 (34.6 - 50.4)**

** Genetic test consent forms were available to complete digitally immediately after patients had either a) viewed all of the digital screens or b) the patient record had been updated following the telephone appointment.*
***Sequencing of the sample was initiated at a minimum of 1 day post genetic test consent to allow time for patients to change their mind.*

Supplementary figure 4: BRCA-DIRECT pathway stages. 1) Pre-test information delivery; 2) decision to proceed with genetic test; 3) testing of sample; 4) results delivery.

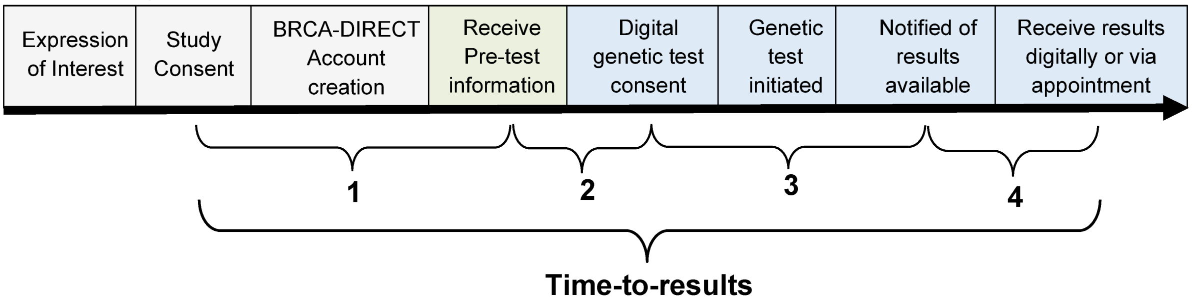

8

Supplementary table 4: Patient reported access and support requirements during involvement with the BRCA-DIRECT digital pathway.

Area of support/access	Responses	All		Pre-test information allocation			
				Digital		Telephone	
		n	%	*n*	%	*n*	%
Devices used to access BRCA-DIRECT	Smartphone only	45	45.0%	24	49.0%	21	41.2%
	Desktop Computer/Laptop only	24	24.0%	12	24.5%	12	23.5%
	Desktop Computer/Laptop, Tablet and Smartphone	1	1.0%	1	2.0%	0	0.0%
	Desktop Computer/Laptop and Smartphone	14	14.0%	2	4.1%	12	23.5%
	Tablet and Smartphone	7	7.0%	4	8.2%	3	5.9%
	Desktop Computer/Laptop and Tablet	1	1.0%	0	0.0%	1	2.0%
	Tablet only	8	8.0%	6	12.2%	2	3.9%
Assistance required with providing sample	Yes: Clinical	12	12.0%	9	18.4%	3	5.9%
	Yes: friend or family member	1	1.0%	1	2.0%	0	0.0%
	No	87	87.0%	39	79.6%	48	94.1%
Technical assistance required to access/use the BRCA-DIRECT platform	Yes: Clinical	2	2.0%	1	2.0%	1	2.0%
	Yes: Friend or family member	5	5.0%	3	6.1%	2	3.9%
	No	93	93.0%	45	91.8%	48	94.1%
Made a call to the hotline (patient reported)	Yes	8	8%	4	8.2%	4	7.8%
	No	92	92%	45	91.8%	47	92.2%

Supplementary table 5: Healthcare professional reported satisfaction with elements of the BRCA-DIRECT digital pathway compared with standard care

I have found the following aspects of the BRCA-DIRECT digital pathway to be equivalent (or superior) to standard care:	Disagree (%)	Neither Agree nor Disagree (%)	Agree (%)	Responses (n)
Biosample collection (ie saliva)	0.0%	36.4%	63.6%	11
Delivery of standardised pre-test information (ie digital)	0.0%	45.5%	54.5%	11
Patient access to individualised specialist advice (ie genetics specialist telephone hotline)	0.0%	36.4%	63.6%	11
Test uptake	0.0%	9.1%	90.9%	11
End-to-end time-to-results	27.3%	27.3%	45.5%	11
Communication of test results to patients	9.1%	18.2%	72.7%	11
Communication of test results to clinicians	27.3%	0.0%	72.7%	11
Psychological effect on patients	18.2%	18.2%	63.6%	11
Use of healthcare professional time	27.3%	0.0%	72.7%	11
Communication of patient status/updates	18.2%	9.1%	72.7%	11

Torr B, *et al. J Med Genet* 2022; 59:1179–1188. doi: 10.1136/jmg-2022-108655

Supplementary table 6: Healthcare professional reported benefits and challenges/short-falls of the BRCA-DIRECT digital pathway

	Benefits (Reported by)	(n)	Challenges (Reported by)	(n)
For the Clinician	Greater access to testing	5	Lack of information regarding patient progression	2
	Reduced clinical time	4	Treatment delays: as a result of laboratory turnaround times	2
	Faster turnaround of results	2	Treatment delays: due to failure of patient to progress or lack of understanding digital process	2
	Simpler pathway/process	4	Difficult to ensure patient comprehension of the genetic testing information	1
For the Patients	Non-invasive procedure	3	Digital literacy/access to digital pathway	5
	Faster turnaround time	5	Treatment delays: as a results of laboratory turnaround times	2
	More empowered	3	Treatment delays: as a result of patient requirement to actively proceed with the pathway.	1
	Simpler pathway	2	Additional test/process to engage with during active treatment or difficult time.	3
	Greater involvement of family and sharing of information	2	Eligibility/language barriers	1
	Greater access to testing	3		

Supplementary table 7: Patient reported feedback on the content of pre-test information delivered either digitally via the BRCA-DIRECT platform (fully-digital pathway) or via a telephone appointment with a genetics specialist nurse or counsellor (partially-digital pathway).

Pre-test information feedback		All		Digital		Telephone	
		n	%	*n*	%	*n*	%
Amount of information	Too much	6	6.0%	4	8.2%	2	3.9%
	Too little	2	2.0%	1	2.0%	1	2.0%
	About right	92	92.0%	44	89.8%	48	94.1%
Information content	Too complicated	5	5.0%	4	8.2%	1	2.0%
	Too simple	0	0.0%	0	0.0%	0	0.0%
	About right	95	95.0%	45	91.8%	50	98.0%

Torr B, *et al. J Med Genet* 2022; 59:1179–1188. doi: 10.1136/jmg-2022-108655

Supplementary table 8: Average (mean) knowledge scores out of a total of 14 points at baseline (T0) and 7-days post-genetic test consent (T1) in all patients, patients randomised to receive pre-test information digitally only (fully-digital pathway), and patients randomised to receive pre-test information via telephone consultation with a genetics professional only (partially-digital pathway).

**Group**	**Baseline (T0)**			**7 days post-genetic test consent (T1)**		
	n	Mean score	SD	n	Mean score	SD
All patients	128	5.2	3.3	114	8.6	3.5
Digital Only	63	4.7	3.1	56	7.3	3.7
Telephone Only	65	5.6	3.4	58	9.9	2.7

Supplementary table 9: Knowledge score questions and number of patients answering individual knowledge questions correctly in the digital and telephone pre-test information arms at baseline (T0) and 7-days post-genetic test consent.

	Digital				Telephone			
	T0		T1		T0		T1	
Number of patients at time point	63		56		65		58	
**Questions**	**Number of patients answering correctly, n (%)**							
I can only inherit gene faults (pathogenic variants) in a BRCA gene from my mother	23	(36.5%)	30	(53.6%)	26	(40.0%)	47	(81.0%)
If my mother has a gene fault (pathogenic variant) in a BRCA gene, I will definitely inherit it	29	(46.0%)	37	(66.1%)	38	(58.5%)	54	(93.1%)
If a woman has a gene fault (pathogenic variant) in a BRCA gene, her daughter is more likely to have inherited the gene fault (pathogenic variant) if the daughter looks more like her mother than her father.	30	(47.6%)	34	(60.7%)	35	(53.8%)	50	(86.2%)
All women with a BRCA gene fault (pathogenic variant) will develop breast cancer	30	(47.6%)	45	(80.4%)	40	(61.5%)	54	(93.1%)
About 30 in every 100 women (30%) who develop breast cancer have a gene fault (pathogenic variant) in BRCA1/BRCA2/PALB2	3	(4.8%)	6	(10.7%)	9	(13.8%)	25	(43.1%)
A woman with breast cancer and a gene fault (pathogenic variant) in BRCA1/BRCA2/PALB2 has a significantly increased risk compared to the general female population of developing the following cancers:								
Another breast cancer	36	(57.1%)	43	(76.8%)	43	(66.2%)	52	(89.7%)
Cervical cancer (cancer of the cervix)	9	(14.3%)	15	(26.8%)	8	(12.3%)	17	(29.3%)
Ovarian cancer (cancer of the ovaries)	21	(33.3%)	33	(58.9%)	33	(50.8%)	47	(81.0%)
Kidney cancer	13	(20.6%)	19	(33.9%)	18	(27.7%)	24	(41.4%)
If my BRCA-test shows I have a gene fault (pathogenic variant), my relatives can be tested to see if they carry the gene fault (pathogenic variant)	42	(66.7%)	44	(78.6%)	48	(73.8%)	56	(96.6%)
If my BRCA-test shows a VUS (variant of uncertain significance), my relatives will routinely be tested to see if they carry the VUS	7	(11.1%)	22	(39.3%)	6	(9.2%)	35	(60.3%)
Female family members found to carry a BRCA gene fault (pathogenic variant) may be offered surgery to reduce their risk of developing cancer	31	(49.2%)	32	(57.1%)	39	(60.0%)	46	(79.3%)
A woman with breast cancer and a gene fault (pathogenic variant) in her BRCA genes can't get life insurance cover	17	(27.0%)	27	(48.2%)	18	(27.7%)	43	(74.1%)
If any relatives without cancer take a BRCA-test and the result shows they have a gene fault (pathogenic variant), they must declare their result if they wish to get life insurance	6	(9.5%)	20	(35.7%)	6	(9.2%)	21	(36.2%)

Torr B, et al. J Med Genet 2022; 59:1179–1188. doi: 10.1136/jmg-2022-108655

Supplementary figure 5: Knowledge test: Percentage of patients answering individual knowledge questions correctly and the overall average (mean) knowledge score for patients (% correct out of a total of 14) in the digital (green) and telephone (blue) pre-test information arms at baseline (T0) (lighter bars) and 7-days post-genetic test consent (T1) (darker bars).

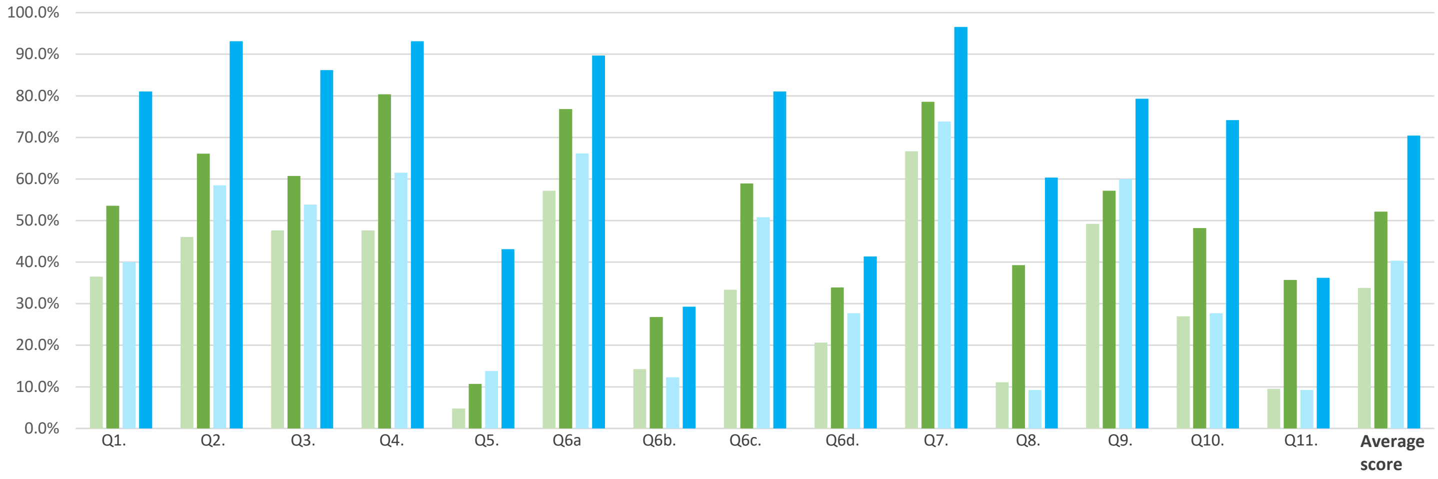

**Q1.**	I can only inherit pathogenic faults in a BRCA gene from my mother (**A**: False)	**Q6.**	A woman with breast cancer and a pathogenic fault in BRCA1/BRCA2/PALB2 has a significantly increased risk compared to the general female population of developing the following cancers:	**Q7.**	If my test for BRCA genes shows I have a pathogenic fault, my relatives can be tested to see if they carry the pathogenic fault (**A**: True)
**Q2.**	If my mother has a pathogenic fault in a BRCA gene, I will definitely inherit it (**A**: False)	**Q6a.**	Another breast cancer (**A**: True)	**Q8.**	If my BRCA gene test shows a VUS (variant of uncertain significance), my relatives will routinely be tested to see if they carry the VUS. (**A**: False)
**Q3.**	If a woman has a pathogenic fault in a BRCA gene, her daughter is more likely to have inherited the pathogenic variant if the daughter looks more like her mother than her father (**A**: False)	**Q6b.**	Cervical cancer (cancer of the cervix) (**A**: False)	**Q9.**	Female family members found to carry a BRCA gene pathogenic fault may be offered surgery to reduce their risk of developing cancer (**A**: True)
**Q4.**	All women with BRCA gene pathogenic faults will develop breast cancer (**A**: False)	**Q6c.**	Ovarian cancer (cancer of the ovaries) (**A**: True)	**Q10.**	A woman with breast cancer and a pathogenic fault in her BRCA genes can't get life insurance cover (**A**: False)
**Q5.**	About thirty in every hundred women (30%) who develop breast cancer have a pathogenic fault in BRCA1/BRCA2/PALB2 (**A**: False)	**Q6d.**	Kidney cancer (**A**: False)	**Q11.**	If any of her relatives unaffected with cancer take a BRCA gene test and the result shows they have a pathogenic fault, they must declare their BRCA gene status if they wish to get life insurance (**A**: False)

Digital T0 | **Digital T1** | **Telephone T0** | **Telephone T1**

Supplementary table 10: Mean Intolerance of Uncertainty, 'trait' anxiety (State Trait Anxiety Index (STAI) Y2) and 'state' anxiety (STAI Y1) scores of all patients and those randomised to receive digital pre-test information or telephone pre-test information only. Mean 'state' anxiety scores (STAI Y1) are captured at baseline (T0), 7-days post-genetic test consent (T1), 7-days post-genetic test results (T2), and 28-days post-genetic test results (T3) and exclude patients who completed >5 days after the time point.

**Cohort**		**Intolerance of Uncertainty**	**Anxiety**				
			Trait	State			
				T0	T1	T2	T3
All	Mean	27.7	39.6	44.5	41.5	37.6	37.7
	SD	9.6	11.3	13.4	14.0	13.2	13.4
	n	128	128	128	117	103	100
Digital	Mean	27.2	39.5	45.1	42.5	37.3	37.7
	SD	9.7	11.6	13.6	13.4	12.9	13.2
	n	63	63	63	56	50	50
Telephone	Mean	28.2	39.7	44.0	40.5	37.5	37.5
	SD	9.5	11.0	13.4	14.6	13.7	13.6
	n	65	65	65	61	53	50

Torr B, *et al. J Med Genet* 2022; 59:1179–1188. doi: 10.1136/jmg-2022-108655

Supplementary table 11: Progression criteria outcomes.

Progression Criterion		Evaluation	Criterion met?
**1**	Recruitment rate during the pilot is >90% of what is required to meet the full study target (1000 patients recruited across two hospital trusts equivalent in case load).	A recruitment rate of 1.3 per day was observed over the study pilot recruitment period.<br><br>To meet the recruitment target of 1000 patients, a recruitment rate of 2.7 would be required in the full study (870 patients recruited with 326 days).<br><br>With the addition of another NHS hospital trust, expected to recruit at equal or greater rate (based on case load), recruitment rate can be predicted to equal 2.6 per day (96.3% of the required rate).	Progression criterion met.
**2**	Retention is >75% at final follow-up.	Retention at final follow up (T3) = 84.6% (110/130 patients).	Progression criterion met.
**3**	>80% of patients complete surveys within a window of 7 days of the expected deadline.	Patient completion of outcome measures within a window of 7-days:<br>T1 = 95.1%;<br>T2 = 91.2%;<br>T3 = 90.9%	Progression criterion met.
**4**	A satisfaction score of ≥3 in at least 80% of patients for questions 9a (How satisfied were you with the way you received your pre-test information?) and 9b (How satisfied were you with the way you received your results?) of the Patient Satisfaction Survey (PSS).	Patient-reported satisfaction of >3 with method of receiving:<br>Pre-test information = 98.0%<br>Results = 95.0%	Progression criterion met.
**5**	Average (mean) knowledge questionnaire score at T1 at least as high as the average at T0 in both arms.	Overall, mean knowledge scores increased from 5.2/14 (SD 3.3) at T0 to 8.6/14 (SD 3.5) at T1.<br><br>Digital arm: T0 = 4.7/14 (SD 3.1) and T1= 7.3/14 (SD 3.7)<br><br>Telephone arm: T0 = 5.6/14 (SD 3.4) and T1 = 9.9/14 (SD 2.7)	Progression criterion met.

Torr B, *et al. J Med Genet* 2022; 59:1179–1188. doi: 10.1136/jmg-2022-108655
