# Supplementary material for: A digital pathway for genetic testing in UK NHS patients with cancer: BRCA-DIRECT randomised study internal pilot
Source: J Med Genet. 2022 Jul 22;59(12):1179–88. doi: 10.1136/jmg-2022-108655 (PMC9691828; doi:10.1136/jmg-2022-108655)
Supplement: Supplementary data [file jmg-2022-108655supp003.pdf]

19/01/2022

Genetics and Cancer risk

Some people are at greater risk of cancer than others. This can be because of genetic factors inherited from their parents.

Usually this is due to inheriting from both parents a greater than average dose of thousands of minor genetic factors. However, sometimes there can be a fault in a particular gene inherited from one parent that causes a large increase in the risk of cancer.

This **gene fault** is called a **'pathogenic variant'** and the **gene in which it is found** is called a **'cancer susceptibility gene'.**

If you have been born with a gene fault (pathogenic variant) in a cancer susceptibility gene, it does not mean that you will definitely develop cancer, but it makes it *more likely* than someone who was not born with the gene fault (pathogenic variant).

Inherited Cancer Risk

These genes are 'cancer susceptibility genes' as they play an important role in controlling cell growth.

If the gene contains a fault (a pathogenic variant), the gene doesn't function properly. This can mean that cells may grow and divide in an uncontrolled fashion leading to cancer.

Three cancer susceptibility genes are strongly associated with an increased risk of developing breast cancer. They are called BRCA1, BRCA2, PALB2, and are often known as the 'BRCA genes'.

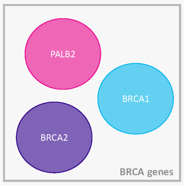

Inherited Cancer Risk (continued)

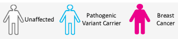

First Breast Cancer Risk

An **average woman in the general population** has a **12 % risk** of developing breast cancer by the age of 80.

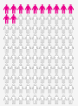

12 out of 100 women will have developed breast cancer by the age of 80.

A woman with a gene fault (pathogenic variant) in BRCA1 or BRCA2, has approximately a **70% risk** of developing breast cancer by the age of 80.

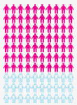

70 out of 100 women will have developed breast cancer by the age of 80.

A **woman with a gene fault (pathogenic variant) in PALB2** has approximately a **50% risk** of developing breast cancer by the age of 80.

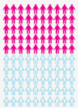

50 out of 100 women will have developed breast cancer by the age of 80.

Inherited Cancer Risk (continued)

Women with breast cancer <u>and</u> a gene fault (pathogenic variant)

Compared to women who do not have a gene fault (pathogenic variant), a woman with a gene fault (pathogenic variant) in one of her BRCA genes, who goes on to develop breast cancer also has:

- a higher risk of developing **another breast cancer** in the same or opposite breast.
- a higher risk of developing **cancer of the ovaries** (or fallopian tubes) - especially for BRCA1
- a small increase in risk of **pancreatic cancer** - BRCA2 and PALB2 only

Male Cancer Risk

BRCA2 is also associated with a modest increase in risk for men of developing **prostate cancer**. BRCA2 and PALB2 increase the risk of **male breast cancer** but it is still uncommon.

Inherited Cancer Risk (continued)

Cancer Risk	The general female population	A woman with a gene fault (pathogenic variant) in:		
		BRCA1	BRCA2	PALB2
**Ovarian Cancer**<br>Average lifetime risk to age 80	1.5%	44%	17%	5%
**Pancreatic Cancer**<br>Average lifetime risk to age 80	1-2%	1-2%	2-5%	2-5%
**First Breast Cancer**<br>Average lifetime risk to age 80	12%	70%		50%
**A second Breast Cancer after the first one**<br>In next 10 years	5%	18%		Not well known

Testing your BRCA genes

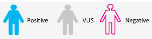

A 'BRCA-test' looks at the DNA in your cells. A sample of your saliva or blood is sent to the laboratory where DNA is removed from the cells. The part of the DNA which contains your BRCA genes is examined to see if it contains genetic changes inherited from your parents.

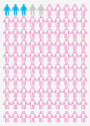

There are three possible test results:

Negative: No gene faults (pathogenic variants) were found in your BRCA genes.

Variant of Uncertain Significance (VUS): In 2 out of 100 tests (2%) we find a VUS. A VUS result means that we currently don't know if the gene change is harmless or if it increases your risk of developing cancer. Overall, most VUSs are likely to be harmless.

Positive: A gene fault (pathogenic variant) known to be associated with increased cancer risk was found in your genes. **3 in 100 women with breast cancer (3%)** will be found to have a gene fault (pathogenic variant) in their BRCA genes.

After receiving your test result, you will be sent a full report and detailed letter. A copy of this letter will be sent to your GP and your cancer clinicians.

It is standard NHS practice to store DNA samples used for genetic testing. Your DNA may be used for 'quality control' including for testing of your other family members. Your test data will be stored securely as part of your NHS clinical record.

1

19/01/2022

Why have a BRCA-test?

The reasons for having a BRCA-test are to get more information about:

- Why you developed cancer
- Which drugs and surgery are best for treating your breast cancer
- Whether or not you are at high risk of developing a new breast cancer
- Whether or not you are at high risk of developing other cancers, such as ovarian cancer
- Whether or not your family members (both male and female) are at increased risk of developing breast and other cancers associated with these genes (ovarian, pancreas, male breast and prostate cancer).

Receiving a test result that is negative

Approximately 95 out of 100 (95%) women with breast cancer will receive a negative result from their BRCA-test.

A negative test result means that:

	It is highly unlikely that your BRCA genes played a part in the development of your breast cancer
	Your risk of developing another new breast cancer is the same as other women your age who have a similar first breast cancer and family history.
	It is unlikely that your close family members are at particularly high risk of breast cancer, unless you have a significant family history of breast cancer.

Receiving a test result that is a VUS

A **variant of uncertain significance (VUS)** is a genetic change that we are unable to clearly interpret. This means that we cannot say categorically whether this gene change is pathogenic (harmful) or harmless. The majority of changes found in genes are harmless and therefore most of these VUSs are likely to be harmless. Around 2 out of 100 BRCA-tests (2%) will return a VUS result.

If your test result shows we have found a VUS:

	We will continue your care as if you had received a negative test result.
	We do not test your family for the VUS. Instead, we manage you and your relatives based on the pattern of breast cancer in the family.
	We may contact you in the future if we receive new information telling us more definitely that your VUS is harmless or is pathogenic (harmful).

What if your test result is positive (gene fault (pathogenic variant) found)?

3 out of 100 (3%) women with breast cancer will receive a positive result from their BRCA-test.

Finding a gene fault (pathogenic variant) in one of your BRCA genes means that:

	This gene fault is likely to have played a part in the development of your breast cancer
	The best drugs to treat your current/recent breast cancer may be different to those used for other breast cancers.
	You are at increased risk of developing another breast cancer in the same or opposite breast. This may influence the options for breast surgery for treating your breast cancer
	You may be at increased risk of developing ovarian cancer and other cancers
	Other members of your family may also carry the same gene fault (pathogenic variant) and be at increased risk of developing cancer

What happens next if your result is positive?

1. You will have a **telephone consultation with a Genetic Counsellor** from the BRCA-DIRECT study to answer any questions you may have.
2. Your **cancer clinicians will talk with you about options** for surgery and/or drugs to treat your breast cancer.
3. You will be **offered an appointment at your local Clinical Genetics service**. Here you can discuss your cancer risk in more detail and the options available to you. They will also advise you about contacting your relatives to offer them genetic testing.

What are the reasons for not having a BRCA-test?

Some women find the information about their breast cancer, and the treatment choices that need to be made, overwhelming.

These women may prefer not to have a BRCA-test at this time.

Other women are concerned about the implications of a genetic test on their health insurance policy.

Screens 15 and 16 provide more information about this.

2

19/01/2022

If I don't want a BRCA-test now, can I have it later?

1. You can have a BRCA-test **through the BRCA-DIRECT study** anytime until it closes.
   - If you decide to have the test later, you may need to supply a new saliva/blood sample and consent form.
   - It is anticipated the study will close in September 2022, but it may close earlier if recruitment is quicker than anticipated.

2. Fewer than 1 in 5 (20%) women with breast cancer are **eligible for standard NHS BRCA-testing**.
   - If you are eligible, you can have an NHS test at any time in the future.

3. Some women choose to **pay for a genetic test from the private sector.** We advise discussion with a trusted knowledgeable health care professional if you are considering this option as some private providers may offer unreliable tests and information.

Additional Information

The following screens contain detailed information on the following:

- Genetic tests and insurance
- Risk reduction
- More about genetics (cancer risk and inheritance)

If you do not understand the information you have already read or have additional questions, please contact the Genetic Counsellor hotline on **+4420 3437 6514 (9am – 5pm, Monday to Friday)**

Genetic tests and Insurance

Insurers routinely request details on your own diagnoses and your family history of disease, and this may influence the terms or premiums. The insurance industry differentiates between a **diagnostic** genetic test and a **predictive** genetic test.

Diagnostic genetic test

This is when you have a genetic test following diagnosis of a condition (e.g. a BRCA-test following a diagnosis of breast cancer).

The Association of British Insurers (ABI) state that diagnostic genetic tests can form part of relevant medical information when applying for a new policy, and therefore must be included in your information. In practice, any new applications for life, critical illness and income protection insurance will be strongly influenced by your recent diagnosis of breast cancer. It is unlikely that terms or premiums will be significantly influenced by information regarding detection of a BRCA gene fault (pathogenic variant).

Genetic tests and Insurance

Insurers routinely request details on your own diagnoses and your family history of disease, and this may influence the terms or premiums. The insurance industry differentiates between a **diagnostic** genetic test and a **predictive** genetic test.

Predictive genetic test

This is when you have **a genetic test for a condition that runs in your family but you currently have no signs of disease** (e.g. a daughter unaffected with cancer having a test to look for a BRCA1 gene fault (pathogenic variant) that was found in her mother with breast cancer).

- A Code of Practice has been developed between the Government and the ABI, which restricts insurers from demanding or using the results of predictive genetic tests.
- If (for example) you are found to carry a BRCA1 gene fault (pathogenic variant) and your sister, unaffected with cancer, takes a predictive test to see if she carries the BRCA1 gene fault (pathogenic variant) found in you, the insurer cannot ask you or your sister about her having the predictive test or about the test result.
- In some cases, it may be in the interests of an unaffected person to declare a negative (normal) result if there is a strong family history of relevant cancers.

What can be done about reducing the risk of developing a new cancer for those found to carry a BRCA gene fault (pathogenic variant)?

New Breast Cancer

The risk of developing a new breast cancer can be reduced by having an operation to remove one or both healthy breasts. This is called **risk-reducing mastectomy**. Various types of breast reconstruction are available following risk-reducing mastectomy.

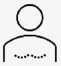

Women with a new diagnosis of breast cancer and a BRCA gene fault (pathogenic variant) may choose to have bilateral mastectomy as the operation for removal of their original cancer. **Female relatives without cancer** carrying the BRCA gene fault (pathogenic variant) may also be offered risk-reducing mastectomy.

Another option is to have very regular scans and/or mammograms with the intention of picking up any new breast cancers at an early stage.

What can be done about reducing the risk of developing a new cancer for those found to carry a BRCA-gene fault (pathogenic variant)?

Ovarian Cancer: can be aggressive with poor outcomes. Women over the age of 40 may be offered an operation to remove their ovaries and the Fallopian tubes (tubes which carry eggs to the womb). This is often done as keyhole surgery, where the surgeon only needs to make a tiny cut to remove the ovaries and fallopian tubes.

Prostate Cancer: men may be offered screening using a combination of blood tests, scans and removal of samples (biopsies) from the prostate to help detect prostate cancer at an early stage.

Pancreatic Cancer: pancreatic screening involves scans or tests using an internal camera; it could be offered to certain carriers of a BRCA-gene pathogenic variant, especially if there is pancreatic cancer in the family.

If you are found to carry a BRCA-gene fault (pathogenic variant) in the BRCA-DIRECT study, you will be referred to clinical genetics where you can discuss your cancer risk and options available to you in more detail.

Torr B, *et al. J Med Genet* 2022; 59:1179–1188. doi: 10.1136/jmg-2022-108655

19/01/2022

More about genetics: Genetics and Cancer risk

All cells in our body contain two identical sets of a blueprint called DNA. DNA looks like a long string and contains sequences of code called genes. Genes are responsible for physical characteristics such as eye colour but they also perform other important functions. We inherit one set of DNA from our mother and one from our father, so our genes come in pairs.

When a cell divides, its DNA is copied into the new cells. Genetic changes can arise by chance as the DNA is being copied. Build-up of genetic changes is a normal process that happens over time in all our cells. Some genetic changes can switch on 'cancerous' behaviour in the cells, where there is loss of control over how the cells divide, grow and spread. 'Environmental' factors, such as UV light and tobacco smoke will increase the rate of genetic changes.

If you have been born with a gene fault (pathogenic variant) in a cancer susceptibility gene, this means that fewer additional genetic changes will be required for your cells to switch to the cancerous behaviour. Therefore, if you have an inherited gene fault (pathogenic variant), it does not mean that you will definitely develop cancer but it makes it *more likely* than for someone who was not born with the gene fault (pathogenic variant).

More about genetics: How are genetic changes inherited?

We each have 2 copies of each gene (one inherited from our mother and one inherited from our father). Each parent only provides one set of their genes to each child. The inheritance of gene changes in most cancer susceptibility genes, including BRCA1, BRCA2 and PALB2, is **autosomal dominant**. So if you carry a gene fault (pathogenic variant) in BRCA1/BRCA2 or PALB2, you could have inherited it from either your mother or your father.

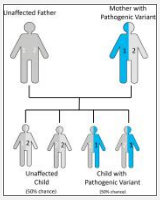

If you carry a gene fault (pathogenic variant), each time you have a child, there is a 50% chance you will pass on the gene fault (pathogenic variant) to the child and a 50% chance that you will not.

This does not mean half your children will inherit the gene fault (pathogenic variant). It may be that all of them or none of them inherit the gene fault (pathogenic variant) (like flipping heads on a coin). If your child has not inherited the gene fault (pathogenic variant), it has 'stopped there' and cannot be passed on to their children.

Genetic traits are inherited independently of each other. Therefore just because a child has inherited the same hair or eye colour as their mother and looks like her, they still have just a 50% chance of having inherited the gene fault (pathogenic variant) she carries.

Where to find more information

If you still have questions about genetic testing or require further information, please contact the BRCA-DIRECT Genetic Counselling hotline on +4420 3437 6514 (9am – 5pm, Monday to Friday).

External Sources of Information
A number of charities and NHS organisations have provided information on genetic testing for breast cancer and BRCA gene testing. We have provided some links below for further information – please note, these are generic resources and some information provided may not be relevant to you or the BRCA-DIRECT study.

- NHS Website https://www.nhs.uk/conditions/predictive-genetic-tests-cancer/
- Royal Marsden NHS Foundation Trust: Beginners Guide to BRCA1 and BRCA2 https://shared-d7-royalmarsden-publicne-live.s3.amazonaws.com/files_trust/s3fs-public/beginners-guide-to-brca1-and-brca2.PDF
- Royal Marsden NHS Foundation Trust: Genetic testing and insurance https://shared-d7-royalmarsden-publicne-live.s3-eu-west-1.amazonaws.com/files_trust/s3fs-public/GE-1460-04.pdf
- Breast Cancer Now https://breastcancernow.org/information-support/have-i-got-breast-cancer/am-i-risk/breast-cancer-in-families/genetic-testing
- Macmillan https://www.macmillan.org.uk/cancer-information-and-support/worried-about-cancer/causes-and-risk-factors/brca-gene
- Prevent Breast Cancer: https://preventbreastcancer.org.uk

Torr B, *et al. J Med Genet* 2022; 59:1179–1188. doi: 10.1136/jmg-2022-108655
